# Photocontrol of the β-Hairpin Polypeptide Structure through an Optimized Azobenzene-Based Amino Acid Analogue

**DOI:** 10.1021/jacs.3c11155

**Published:** 2024-01-16

**Authors:** Raffaella Parlato, Jana Volarić, Alessia Lasorsa, Mahdi Bagherpoor Helabad, Piermichele Kobauri, Greeshma Jain, Markus S. Miettinen, Ben L. Feringa, Wiktor Szymanski, Patrick C. A. van der Wel

**Affiliations:** † Zernike Institute for Advanced Materials, University of Groningen, Nijenborgh 4, 9747 AG Groningen, The Netherlands; ‡ Stratingh Institute for Chemistry, University of Groningen, Nijenborgh 7, 9747 AG Groningen, The Netherlands; § Department of Theory and Bio-Systems, Max Planck Institute of Colloids and Interfaces, 14424 Potsdam, Germany; ∥ Computational Biology Unit, Departments of Chemistry and Informatics, University of Bergen, 5020 Bergen, Norway; ⊥ Medical Imaging Center, University Medical Center Groningen, Hanzeplein 1, 9713 GZ Groningen, The Netherlands

## Abstract

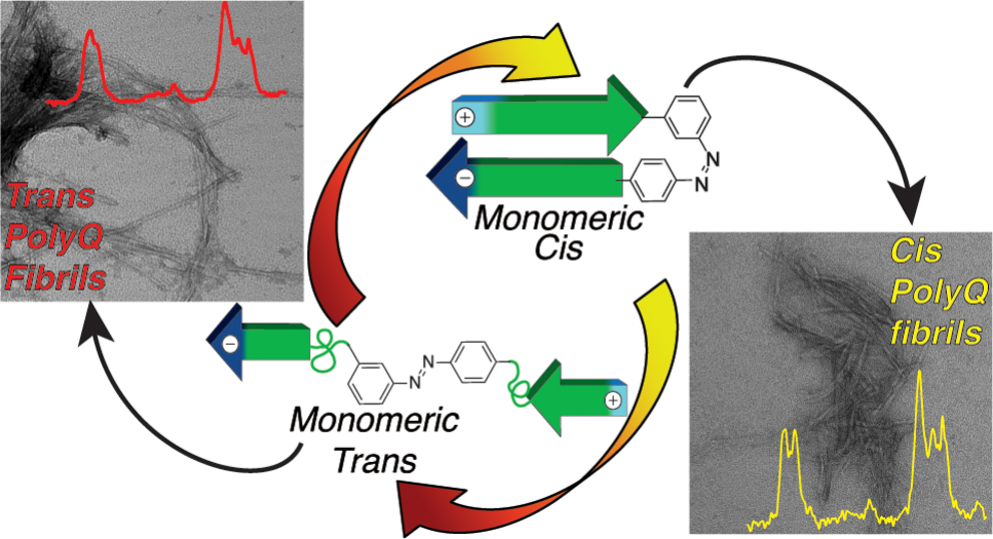

A family of neurodegenerative diseases, including Huntington’s disease (HD) and spinocerebellar ataxias, are associated with an abnormal polyglutamine (polyQ) expansion in mutant proteins that become prone to form amyloid-like aggregates. Prior studies have suggested a key role for β-hairpin formation as a driver of nucleation and aggregation, but direct experimental studies have been challenging. Toward such research, we set out to enable spatiotemporal control over β-hairpin formation by the introduction of a photosensitive β-turn mimic in the polypeptide backbone, consisting of a newly designed azobenzene derivative. The reported derivative overcomes the limitations of prior approaches associated with poor photochemical properties and imperfect structural compatibility with the desired β-turn structure. A new azobenzene-based β-turn mimic was designed, synthesized, and found to display improved photochemical properties, both prior and after incorporation into the backbone of a polyQ polypeptide. The two isomers of the azobenzene-polyQ peptide showed different aggregate structures of the polyQ peptide fibrils, as demonstrated by electron microscopy and solid-state NMR (ssNMR). Notably, only peptides in which the β-turn structure was stabilized (azobenzene in the *cis* configuration) closely reproduced the spectral fingerprints of toxic, β-hairpin-containing fibrils formed by mutant huntingtin protein fragments implicated in HD. These approaches and findings will enable better deciphering of the roles of β-hairpin structures in protein aggregation processes in HD and other amyloid-related neurodegenerative diseases.

## Introduction

**Figure 1:**
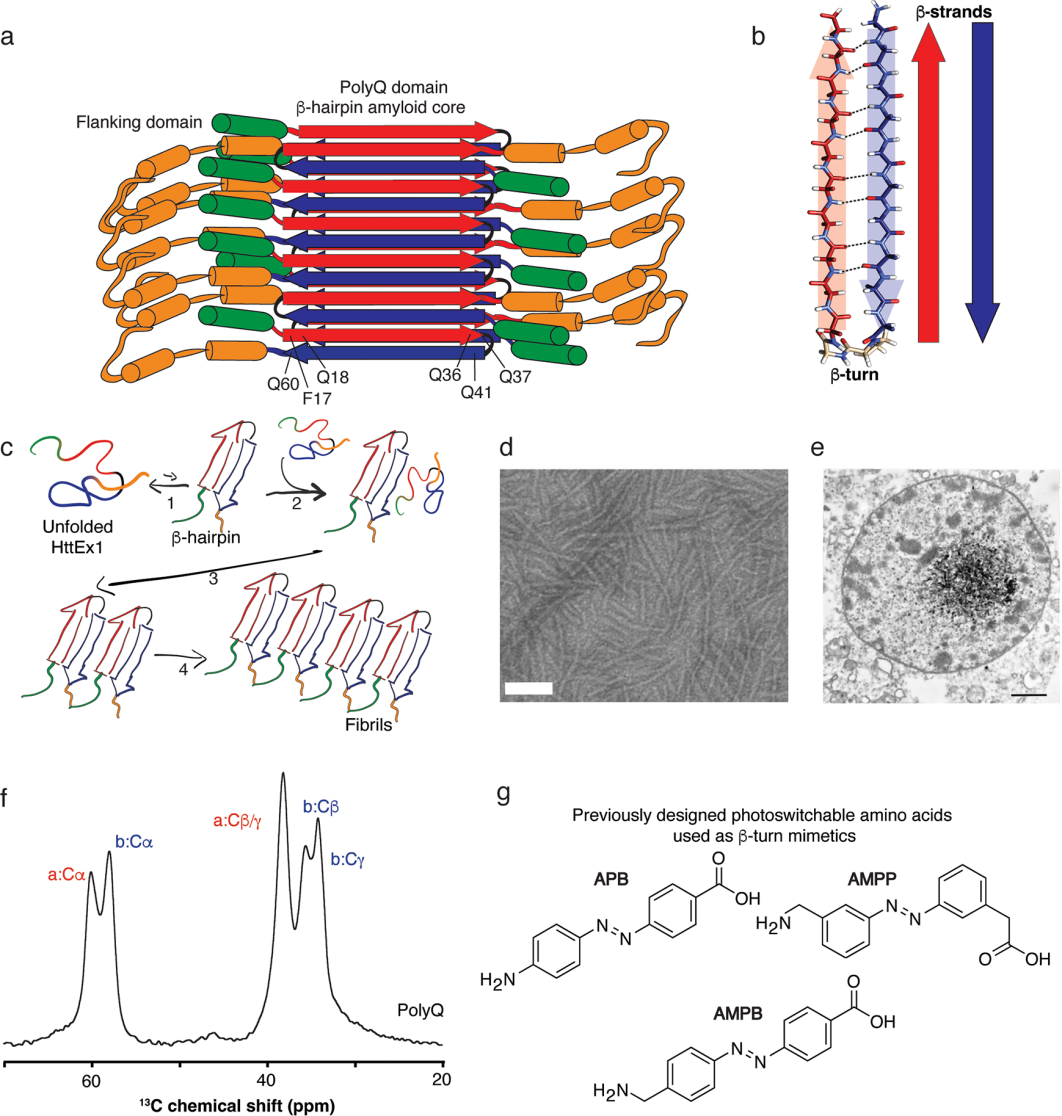
Overview of Huntington’s disease polyQ protein fibril structure and previous azobenzene-based β-turn mimics. (a) Schematic model of the mutant HttEx1 fibril with the β-hairpin polyQ core shown as alternating red and blue arrows and surface-facing flanking domains in orange and green. (b) Schematic model of the polyQ β-hairpin, showing alternating type “a” and “b” β-strands (red and blue, respectively) in the antiparallel polyQ β-sheets. Intramolecular hydrogen bonds and the β-turn are marked. (c) Schematic representation of the fibrillization process in sequential steps: β-harpin formation, recruitment of other monomers, and elongation. (d) Transmission electron microscopy (TEM) image of mutant HttEx1 fibrils (scale bar: 100 nm). (e) Huntingtin aggregates in neuronal cells of HD patient. (f) 1D ^13^C ssNMR spectrum (aliphatic region) of a labeled Gln in polyQ amyloid fibrils. Characteristic glutamine type “a” and “b” signals (red and blue labels) correspond to the alternating β-strands color coded in panels (a, b). (g) Previously reported azobenzene-based amino acids (**APB, AMPB**, and **AMPP**) used to control the β-hairpin formation.^21,28−31^ Panel (b) was reprinted from the Journal of Molecular Biology, vol. 429, Kar et al., Backbone Engineering within a Latent β-Hairpin Structure to Design Inhibitors of Polyglutamine Amyloid Formation, pp. 308–323, Copyright (2017), with permission from Elsevier.^33^ Panel (e) was reprinted from DiFiglia et al. *Science***1997**, 277 (5334), pp. 1990–1993, with permission from AAAS.^34^

Protein aggregation plays a pivotal role in a wide variety of neurodegenerative disorders, such as Huntington’s (HD), Alzheimer’s (AD), and Parkinson’s disease.^1−3^ HD is caused by an expansion of CAG repeats in exon 1 of the gene encoding the huntingtin protein, resulting in an expansion of its polyglutamine (polyQ) region.^3,4^ Aberrant splicing and protein cleavage yield huntingtin exon 1 (HttEx1) fragments prone to aggregation into β-sheet-rich amyloid or amyloid-like fibrils.^4−6^ Notably, mutant HttEx1 fibrils contain a β-hairpin conformation inaccessible to wild-type nonpathogenic proteins (Figure 1a,b).^5,7−9^ In this β-hairpin conformation, two antiparallel β-strands, connected by a short β-turn region, are engaged in intramolecular hydrogen bonding.^10−12^ It has been argued that β-hairpin formation is an early nucleation event in HD, relevant to the onset of aggregation (and thus the disease)^7,13^ (Figure 1c).^5,14^ The interest in these early mechanistic steps stems from a desire to block or redirect pathogenic protein aggregation in HD and other protein aggregation diseases.^5,7,15,16^ Rational design of intervention strategies requires a detailed molecular understanding of the complexities of the aggregation process.^17^ The end product of the aggregation pathway can be studied, by performing electron microscopy (EM) (Figure 1d,e) and solid-state NMR (ssNMR; Figure 1f), to give a molecular view of the fibril and its internal structure.^18^ Unfortunately, direct experimental dissection of the complex aggregation pathway itself is very challenging. Of particular value would be an approach that can test the hypothesized role of β-hairpin folds at different stages of the aggregation process. Prior studies have shown the use of β-hairpin-stabilizing mutations to study aggregation kinetics as well as aggregate stability.^7,19^ However, such an approach using persistent β-hairpin propensity modulators lacks the temporal control that is necessary to truly dissect the multistep mechanism. For instance, it is desirable to be able to distinguish the role of β-hairpins in the late versus early stages of aggregation, especially given the hypothesized role of β-hairpins in certain preamyloid oligomers. Normal mutations are present at all stages of the aggregation process, complicating the ability to distinguish their impact on the prenucleation ensemble, nucleation, oligomer formation, and elongation processes. Toward such a goal, one can envision a unique strategy that places the hypothesized β-hairpin folding under external dynamic control by introducing light-responsive β-hairpin-(de)stabilizing motifs that can be switched between favoring and disfavoring the β-hairpin fold. In particular, here, we set out to insert a photocontrolled molecular β-turn mimic into the peptide backbone. Previously, azobenzene photoswitches were employed in attempts to probe the role of β-hairpins in the aggregation of other amyloidogenic peptides.^20−24^ Azobenzenes are light-responsive molecules that change their shape and properties upon light irradiation, by interconverting between two isomers: *cis* and *trans*.^25−27^ Several attempts at designing light-sensitive β-hairpin folds employed azobenzene-based amino acids placed into the polypeptide backbone.^21,28,29^ These azobenzene modules varied in their design, featuring either *para*, *para*- (**APB** and **AMPB**)^28−30^ or *meta, meta*-substituted (**AMPP**)^21^ azobenzene cores (Figure 1g). Conceptually, the *trans* configuration has a β-hairpin-disrupting conformation by rotating the surrounding β-strands away from each other. Conversely, the *cis* isomer simulates β-hairpin formation by encouraging the intramolecular hydrogen bonding of the neighboring residues. However, the application of these existing turn mimics to amyloidogenic aggregation studies was limited by their inability to fully replicate the spatial arrangement of a true β-turn as well as their limited photochemical properties (Table S1).^21,28,31^ For instance, **APB** has an overall rigid structure that is not easily accommodated in a stable β-hairpin while also having a very short half-life of 10 min (at 22 °C in DMSO).^20,28^ Such a short half-life limits its usability in biophysical and mechanistic studies that can take hours to complete.^28^ The **AMPB** design allows for higher flexibility due to the presence of a methylene linker, thus allowing more freedom for the attached peptide chain to adopt a proper turn structure (Figure 1g). Nevertheless, **AMPB** exhibits relatively low switching efficiency, with photostationary state distribution (PSD_365 nm_) ratios in the range of 56–67% of *cis* formation under irradiation with 365 nm light, resulting in a mixture of states that prohibits full control over aggregation processes.^29,30^ Furthermore, according to previous studies, **AMPB** is not able to stabilize the β-harpin conformation.^20^ Finally, the **AMPP** has improved photochemical properties in comparison to the above-mentioned azobenzenes, such as a good PSD_365 nm_ and long half-life. However, while the *meta, meta* substitution pattern would seem to support a β-turn in the *cis* isomer, as much as 50% of the photosensitive peptides did not assemble into a β-hairpin structure in prior work.^21,32^ Therefore, even the **AMPP** system seemed to be unsuitable for our polyQ studies, given that it could not fully facilitate amyloid-β β-hairpin assembly in its *cis* form.^21,23^ It is worth noting that limitations of photoswitches become amplified in studies of self-assembly or protein aggregation processes, in which small imperfections can not only redirect a polymorphic aggregation process but would also be multiplied many times in each fibril. Thus, small defects can have big impacts on the resulting fibrillar structure. The imperfect properties of currently available azobenzene-based amino acid β-turn mimics have inspired us to pursue a better photoswitch design that would feature a higher PSD, a longer half-life, and closer mimicking of the desired β-turn structure.

**Figure 2:**
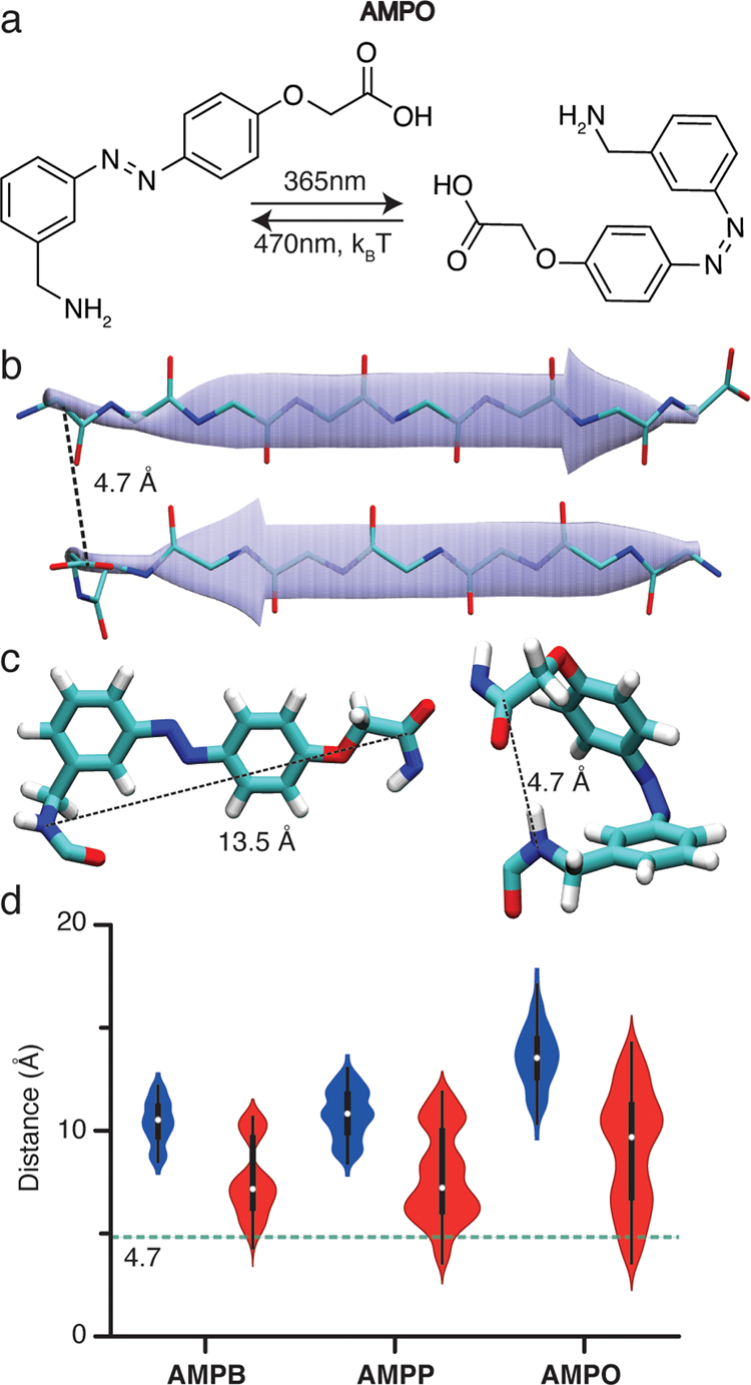
New azobenzene-based amino acid, **AMPO**. (a) Switching between *trans***AMPO** and *cis***AMPO** configurations with UV and visible light. (b) Antiparallel β-sheet amyloid-like structure for a fragment peptide from amyloid-β, depicting the distance (dashed line) between the β-strands that needs to be bridged by a β-turn mimic. (c) 3D model of **AMPO** illustrating the C–N distances (dashed line) in both configurations: *trans* (left) and *cis* (right). (d) Violin plots of C–N distances of all conformers obtained for the *trans*- (blue) and *cis*- (red) isomers of **AMPB**, **AMPP**, and the proposed new design (**AMPO**). The dashed line indicates the distance of 4.7 Å required for the formation of the β-hairpin motif (see also Figure S2 in the SI)

Therefore, we introduce here a new azobenzene-based amino acid for controlling β-turn and β-hairpin formation with light: 2-(4-(3-aminomethylphenylazo)phenoxyacetic acid (**AMPO**, Figure 2a). **AMPO** more efficiently mimics the β-turn distance and orientation and exhibits improved photochemical properties, such as half-life and PSD_365 nm,_ compared to previously reported systems (Figure 1g). As a showcase for **AMPO**, we used it to stabilize or disfavor (depending on the switch state) a β-turn in a polyQ polypeptide, representing a model for the amyloidogenic proteins in HD and other CAG repeat diseases.^5,7^ Using UV–vis spectroscopy and liquid-state NMR, we evaluated, for both the azobenzene photoswitch itself and the **polyQ-AMPO** peptide, the half-life, switching ability, quantum yield, PSD, and resistance to fatigue. EM and ssNMR studies on fibrillar aggregates, formed by *cis* and *trans* configurations, enabled a closer examination of their molecular structure and comparison to the disease-relevant polyQ protein aggregates. Notably, only the *cis* isomer of **AMPO** is found to fully reproduce the structure of the latter, indicating its high structural compatibility with the polyQ β-hairpin fold.

## Results and Discussion

### Design of AMPO

The geometric requirements of a β-turn in a polyQ amyloid context were determined by analysis of known amyloid structures. According to polyQ amyloid studies,^9,35^ β-sheets in the fibrils have an antiparallel structure with a strand–strand distance of ∼4.7 Å (Figure 2b).^1^ To determine the necessary geometry and size required for connecting such strands into a β-hairpin conformation, PDB entries of amyloid-like peptide crystal structures with analogous antiparallel architectures (Figure 2b) were analyzed (Figure S2).^36^ The new azobenzene-based amino acid, **AMPO**, was designed to better fit this spatial arrangement while also featuring improved photochemical properties. Introducing an oxygen atom directly connected to the azobenzene ring in the *para* position relative to the azo bond was envisioned to significantly enhance the photochemical properties of the new β-turn mimic (Figure 2a), namely higher PSD ratios and higher quantum yields, all while retaining sufficiently long half-lives, as observed previously in other examples.^37−39^

Based on molecular modeling studies of candidate azobenzene variants (Figure 2c,d), we chose the *meta*, *para*-substitution pattern as it would be the most beneficial for the formation of a β-hairpin in the *cis* state. The design was guided by conformational search studies on **AMPB**, **AMPP**, and **AMPO**, which were carried out using MacroModel with no constraints (Figure 2c, see the Supporting Information (SI) for details). Subsequently, the C–N end-to-end distance was measured for all conformers, and the distributions were visualized as violin plots (Figure 2d). The computational results indicated that the *meta*, *para*-substitution pattern of **AMPO** would allow the formation of a β-hairpin in the *cis* form, by covering a broader range of C–N distances compared to the previous designs (Figure 2c).^21^ On the one hand, the *cis* configuration was expected to more comfortably adopt the 4.7 Å distance needed for the β-hairpin. Moreover, the *trans* isomer is farther from this distance and would thus be expected to be better at preventing β-hairpin formation.

### Synthesis of AMPO

**Figure 3:**
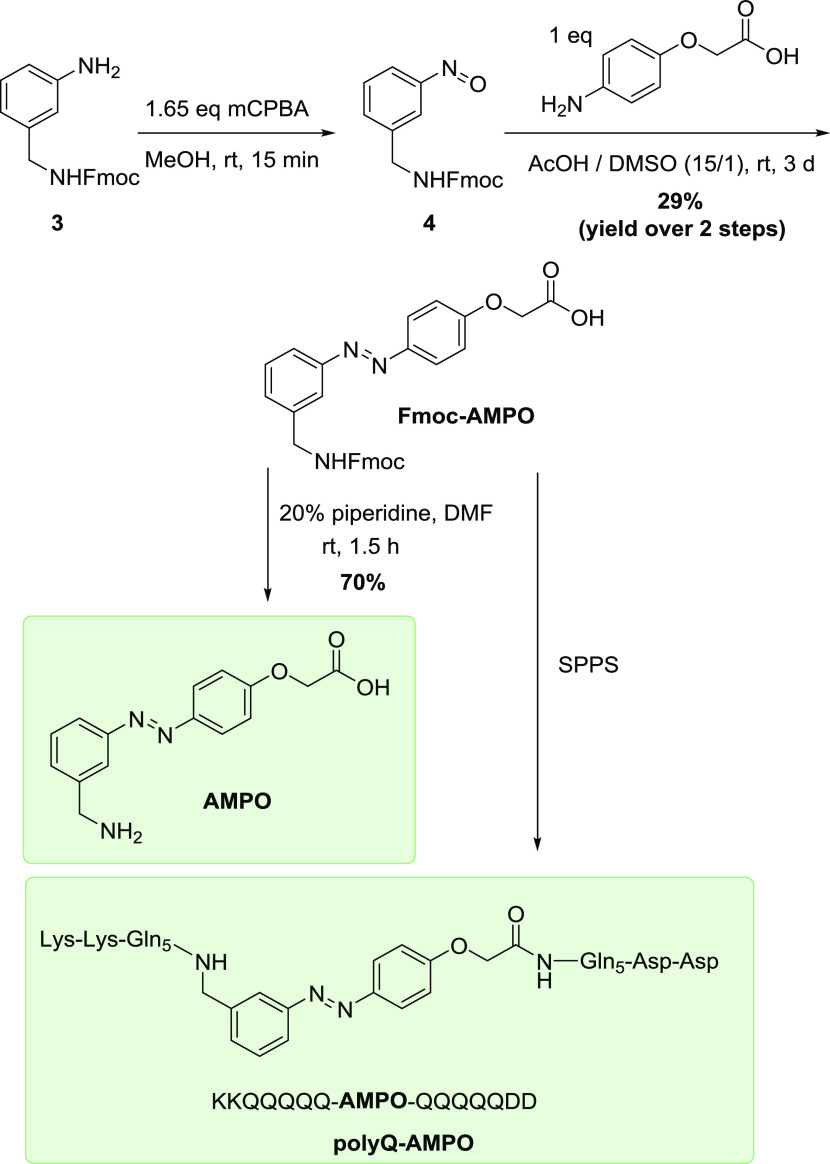
Synthesis of the Fmoc-protected azobenzene-based amino acid **Fmoc-AMPO**.

The solid-phase peptide synthesis-compatible building block **Fmoc-AMPO** can be conveniently synthesized in two steps from Fmoc-protected aniline **3**, by its oxidation (*m*CPBA in methanol) to the nitroso compound **4** and its immediate use in the Baeyer–Mills reaction with the commercially available 2-(4-aminophenoxy)acetic acid (Figure 3). After characterization, **Fmoc-AMPO** was further used for solid-phase peptide synthesis to obtain **polyQ-AMPO**, a peptide designed to contain five glutamine (Gln) groups on each side of the photoswitch, to serve as a model system to study polyQ aggregation. PolyQ peptides with low numbers of Gln residues are known to undergo slower aggregation compared to long polyQ but nonetheless can form similarly structured amyloid-like aggregates, especially at higher peptide concentrations.^40^ Two positively charged amino acids (Lys) on the N-terminal end of **polyQ-AMPO** and two negatively charged amino acids (Asp) on the C-terminal end were introduced to aid the β-hairpin formation in the *cis* isomer and increase the overall solubility, as is common in model polyQ peptides.^7,41,42^**Fmoc-AMPO** was also subjected to piperidine-mediated deprotection to **AMPO**, which was further used as a model compound for the photochemical characterization and comparison with the **polyQ-AMPO**.

### Photochemical Properties of AMPO

**Figure 4:**
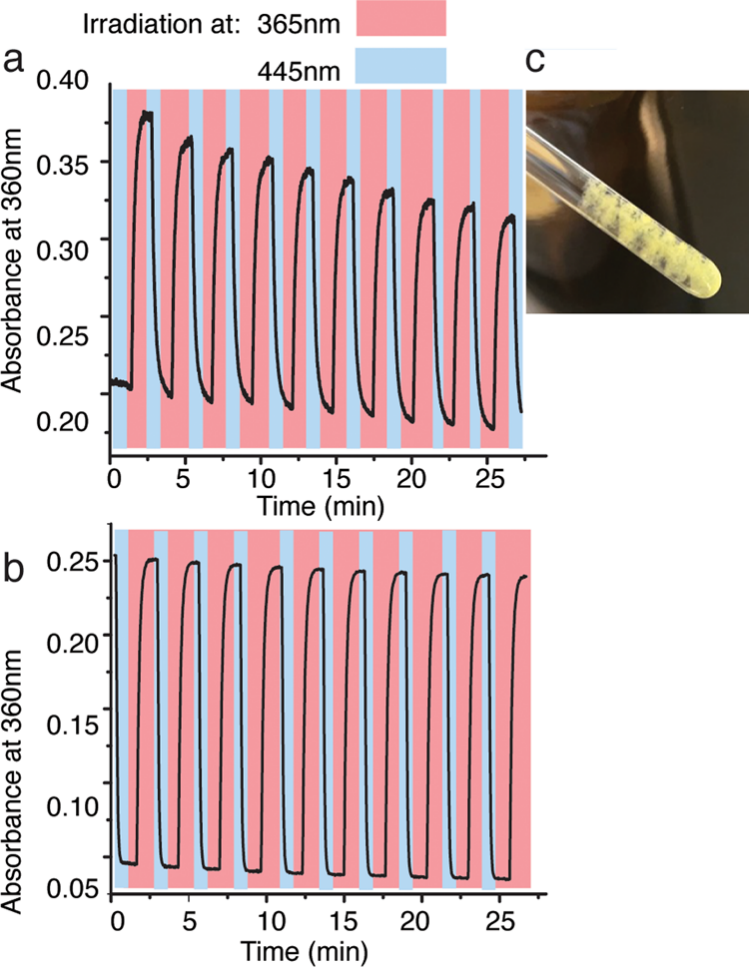
(a) Fatigue resistance test of **polyQ-AMPO** in 9% MeOH and water (45 μM) before the disaggregation protocol (see text). (b) Fatigue resistance test of **polyQ-AMPO** in 9% MeOH and water (45 μM) after the disaggregation protocol.^24,44^ (c) 0.5 mg of **polyQ-AMPO** in 400 μL of MeOD (D_2_O and TFA added) after 3 days.

The photochemical properties of the Fmoc-protected azobenzene **Fmoc-AMPO**, deprotected azobenzene **AMPO**, and the polyQ chain with the azobenzene moiety in the backbone (**polyQ-AMPO**) were determined in different solvents (Table S1). Since we anticipated that the solubility and the photochemical properties of **Fmoc-AMPO** and **AMPO** would be different in different solvents at different pHs, the photochemical properties were studied in a set of different conditions, namely in DMSO (Figure S3), MeOH (Figure S4), and K_2_CO_3_ aqueous solution (Figure S5) for **Fmoc-AMPO**, while the deprotected **AMPO** was tested in MeOH and TFA (Figure S6), K_2_CO_3_ aqueous solution (Figure S7), and water and TFA (pH 2, Figure S8). Finally, the **polyQ-AMPO** peptide was tested in MeOH and water (Figure S9) and in PBS and DMSO (Figure S10). All compounds exhibited extremely high PSD ratios (over 90% *cis*) upon irradiation with 365 nm light, except for **AMPO** in MeOH and TFA (85% *cis*) because of its shortened half-life due to protonation of the azo bond. We observed that high and low pH had a significant effect on the half-life of both Fmoc-**AMPO** and **AMPO**, starting from a range of >98 h for the *cis* isomer (for **AMPO** at pH 8) to 2 min (for **Fmoc-AMPO** at pH 8). These results suggested that pH needs to be taken into consideration for the specific conditions for polyQ aggregation studies. Furthermore, both **Fmoc-AMPO** and **AMPO** exhibited high quantum yields (0.48–0.74) for the forward switching under all tested conditions. Both the quantum yields (experimental procedure in the SI) and the PSD ratios in all tested conditions were substantially higher compared to those of the previously reported azobenzene amino acids (**APB**, **AMPB**, and **AMPP**). The quantum yield at 365 nm irradiation of **polyQ-AMPO** was significantly lower than the reported values for **Fmoc-AMPO** and **AMPO** (Table S1). This reduction of the quantum yield upon attachment of peptide chains is in line with previous reports for related systems.^20,43^ Lastly, the fatigue resistance of **polyQ-AMPO** was tested in 9% MeOH in water (Figure 4a) to observe a consistent drop of absorbance minima and maxima, which is likely caused by aggregation. To reduce the aggregation by increasing the solubility of **polyQ-AMPO**, the sample was pretreated with TFA and HFIP using a disaggregation protocol, which was introduced to remove pre-existing aggregates from the samples.^21,22,43^ The treatment with the disaggregation protocol resulted in an almost negligible absorbance drop during the fatigue studies (Figure 4b).^23,24,44^

### PolyQ-AMPO Peptide Fold in the Monomeric State

**Figure 5:**
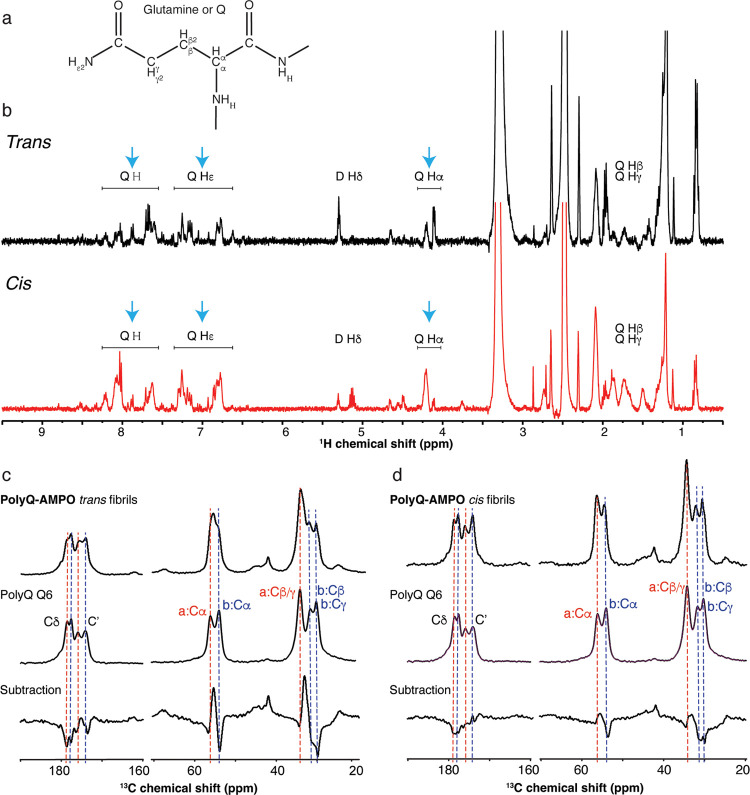
Solution and solid-state NMR spectra of **polyQ-AMPO** monomers and aggregates. (a) Glutamine chemical structure with carbon and hydrogen nomenclature indicated. (b) ^1^H 1D solution-state NMR spectra of both *cis* and *trans* configurations of **polyQ-AMPO**: *trans* (in black in the top) and *cis* (in red in the bottom). Both samples were measured at room temperature at 1 mM concentration in deuterated PBS buffer with 0.5% deuterated DMSO. The labels indicate exchangeable proton (H and Hε) and not exchangeable proton (Hα, Hβ, Hγ, and Hδ), as shown in (a). The blue arrows indicate the difference in proton peak intensity, as further discussed in the text. (c) 1D ^13^C CP MAS ssNMR on unlabeled **polyQ-AMPO** aggregates with the *trans* configuration (top), labeled polyQ (polyQ Q6) in the middle, and *trans* minus polyQ subtraction in the bottom. (d) Analogous ssNMR data for unlabeled **polyQ-AMPO** aggregates with the *cis* configuration (top), labeled polyQ (polyQ Q6), and *cis* minus polyQ subtraction (bottom). The two conformers of the polyQ amyloid are marked with red (form a) and blue (form b) lines.

Next, we examined the conformation of the monomeric azobenzene-containing **polyQ-AMPO** peptide with liquid-state NMR spectroscopy. **PolyQ-AMPO** was HFIP-treated and dissolved in an aqueous buffer (PBS, pH 7, Figure 5a). 1D ^1^H NMR spectra were recorded on two samples: the first sample contains 99% of *trans* isomer; it is denoted as *trans* and it was the thermally equilibrated sample; the second sample is denoted as *cis* as it was irradiated at 365 nm (PSD_365 nm_). As can be seen in the NMR spectra (Figure 5b), the two configurations show different glutamine signals for both the backbone and side chains, indicating the presence of different structures. The *trans* isomer shows two populations for the glutamines’ (Q) α protons (H_α_), suggesting two different conformations (Figure 5b, blue arrows). In addition, the (exchangeable) amide and glutamine side chain epsilon protons (H and H_ε_) have low signal intensities, which indicates fast exchange with the solvent (Figure 5b, blue arrows). In contrast, the *cis* isomer shows a more homogenous structure, as the glutamine α protons (H_α_) are represented by a single dominant peak (Figure 5b, blue arrows). Furthermore, the signals from the amide and glutamine side chain epsilon protons (H and H_ε_) are higher in intensity, suggesting a reduced hydrogen exchange with the solvent, potentially because of a more compact conformation (Figure 5b, blue arrows). According to these results, we observe a clear conformational switch of the peptide between a more-open *trans* configuration of **polyQ-AMPO** and a more closed and also more uniform conformation for the *cis* counterpart.

### PolyQ-AMPO Peptide Conformation in the Aggregated State

A key challenge for in-depth solution-state NMR analysis is the propensity of these peptides to form aggregates, which are invisible to solution-state NMR. To further investigate the aggregates formed by *cis* and *trans* isomers of **polyQ-AMPO**, magic angle spinning (MAS) ssNMR (Figure 5c,d) was performed on these unlabeled (natural abundance) samples. First, the samples were studied by using cross-polarization (CP)-based MAS ssNMR (Figure 5c,d). CP-based experiments reveal signals from rather rigid and immobilized molecules and thus reveal the signals from aggregated, rather than soluble, fractions of the sample.^18,45^ The 1D ^13^C CP-based spectra (Figure 5c,d) show a similar pattern for the aggregated *cis* and *trans* isomers. The chemical shift values and their assignment are reported in Table S2. The assignments stem from a comparison to the prior ssNMR studies of (labeled) polyQ peptide and protein samples. Figure 5 also shows the signals from a polyQ peptide aggregate ([U–^13^C,^15^N-Q6]-Q_30_;^33^ polyQ Q6; Figure 5c,d middle spectrum), in which a single Gln was labeled with ^13^C and ^15^N.^5,9^ Notably, the recorded 1D CP spectra on the *cis***polyQ-AMPO** sample suggest that the fibril core consists predominantly of the same ssNMR signals as observed in previous studies of polyQ aggregates.^7,9^ These specific doubled signals are characteristic hallmarks of the polyQ amyloid state.^7^ They are indicated as “a” and “b” peaks (marked with red and blue lines in Figure 5c,d, respectively) and stem from distinctly structured β-strands in antiparallel β-sheets.^7,9^

These two conformers can be easily detected in the control and in the *cis* sample (Figure 5d, top and middle spectrum) but are not as easily identified in the *trans* configuration (Figure 5c, top spectrum). To better investigate potential differences in the peak position and intensity, the **polyQ-AMPO** spectra were subtracted from the polyQ Q6 spectra. The subtraction performed on the *trans***polyQ-AMPO** species shows that the *trans* isomer fails to fully replicate the normal polyQ amyloid signal (Figure 5c, bottom spectrum). On the other hand, the *cis***polyQ-AMPO** difference spectrum is much smaller, consistent with a close match to the normal polyQ amyloid signal (Figure 5d, bottom spectrum). These differences between the two samples (*cis/trans*) are most easily analyzed based on the Cα peaks, given the more extensive overlap of the signals from the other carbon sites. As seen by ssNMR, the *cis* configuration and the polyQ core of typical polyQ protein fibrils have the same fingerprint. Thus, as per our design aims, the *cis* configuration is geometrically accommodated into the polyQ architecture, similar to previously reported nonswitching β-hairpin turn stabilizers,^7,9,33^ while the *trans***polyQ-AMPO** is unable to fully replicate the polyQ structure, yielding a different fibril structure. The CP ssNMR results denote that the **polyQ-AMPO** fibril core has a highly rigid, ordered structure, with high similarity to normal polyQ aggregates.^9^ The rigidity of both types of aggregates can be concluded from the strong signal intensities observed for this unlabeled material. The lack of flexible components in the samples is further supported by a lack of signal in the 1D ^13^C INEPT-based ssNMR spectra that are selective for highly dynamic molecules (Figure S11). Subtle differences in the structural order of the aggregates are also detected in 2D CP-based heteronuclear correlation (HETCOR) ssNMR spectra (Figure S12). In these 2D experiments, we observed more well-defined peaks for *cis* aggregates, with more peak broadening for the *trans* counterparts (Figure S12a,b), consistent with our 1D ssNMR analysis. This suggests that both aggregates are rigid and ordered but that the degree of order is higher in the *cis* aggregates (Figure S12e,f). Also, this finding is consistent with a better incorporation of the geometry of the new **AMPO** design into the native fibril structure as per our design goals.

**Figure 6:**
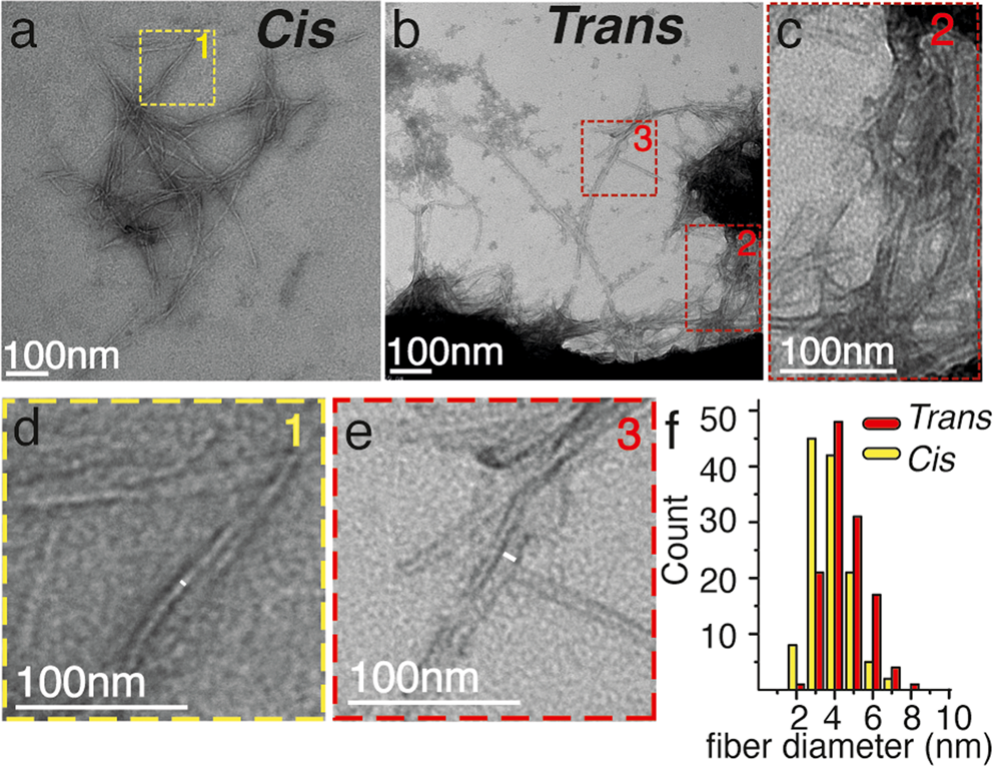
TEM micrographs of aggregates of both **polyQ-AMPO** isomers: (a) *cis* and (b) *trans* isomer. (c) Enlarged area from panel (b) (red box n.2) showing bundled aggregates. (d) Enlarged area from panel (a) (yellow box no. 1) showing a fibril with a width of ∼3 nm. (e) Enlargement from panel (b) (red box n.3) showing a fibril width of ∼6 nm. (f) Fiber width distribution of the *cis* (yellow) and *trans* (red) isomers.

To further examine the two types of aggregates, negative-stain transmission electron microscopy (TEM) was performed (Figures 6, S13 and S14). Both configurations yield peptide aggregates with a fibrillar morphology (Figure 6a–c), as expected for amyloid fibrils formed by polyQ proteins and peptides. However, the two isomers assemble into fibrils with different diameters (Figure 6d–f). Thus, also, the EM-observed morphology reveals a systematic difference between the *cis* and *trans***polyQ-AMPO**-containing fibrils.

### Modeling the Aggregated Peptide Fold

**Figure 7:**
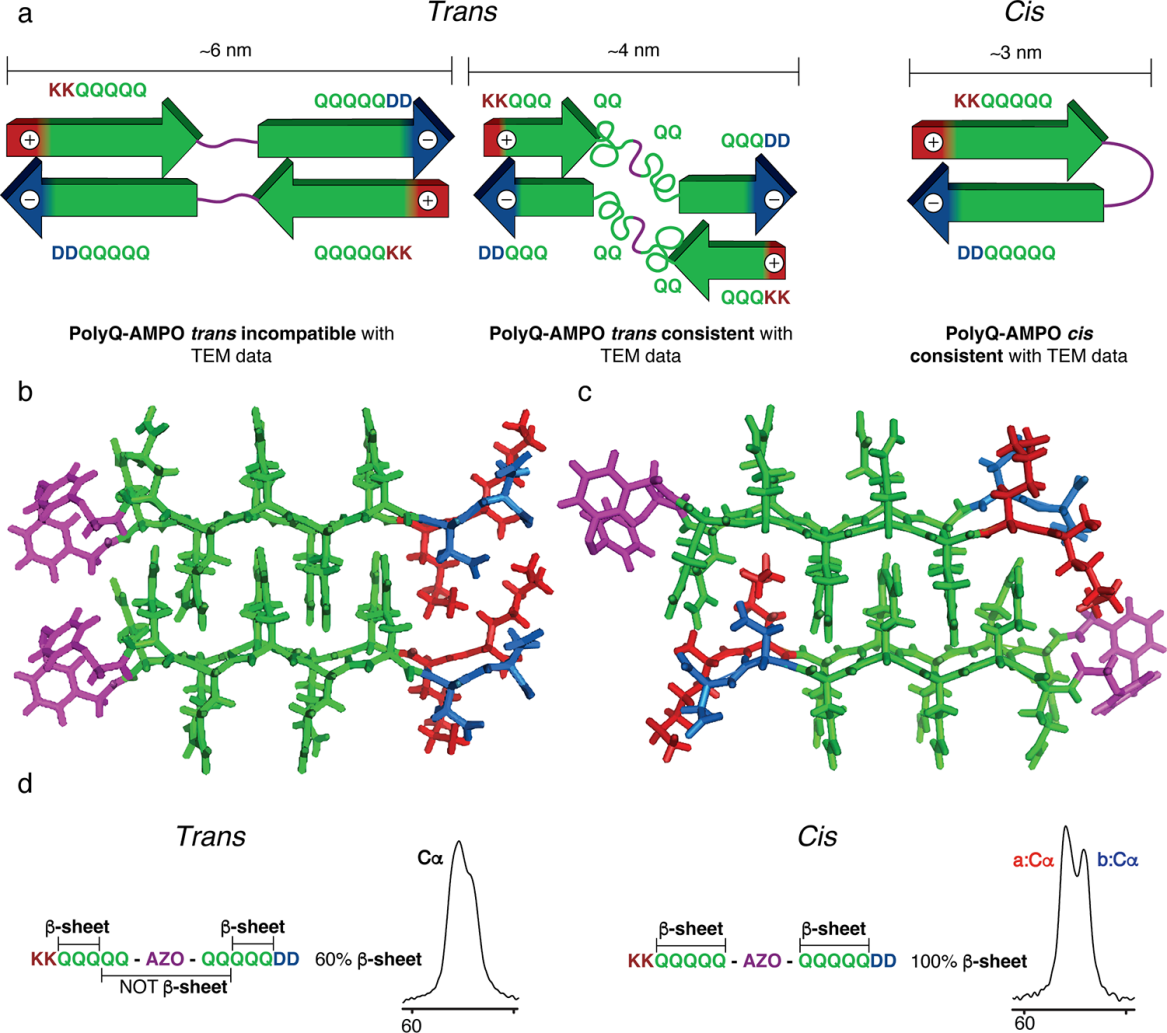
Schematic model of *cis* and *trans***polyQ-AMPO** and 3D model of *cis***polyQ-AMPO** fibrils. (a) Two schematic models of *trans* (left) and schematic model of *cis* (right): a first model of ∼6 nm and incompatible with the TEM data (left), a second model of ∼4 nm fitting with TEM data (middle), and a third model representing the structure of the *cis* isomer (right). (b) Possible 3D model of the *cis***polyQ-AMPO** fibril conformation according to molecular modeling. In this model, the **AMPOs** are located on one side of the polyQ fibril. (c) Another possible model of *cis***polyQ-AMPO** fibrils featuring the **AMPO** alternating on both sides of the fibril. (d) Overview of the two isomers’ structure and the compatibility with the experimental data. The *trans* isomer is characterized by a broad single peak in the Cα region, suggesting a heterogenous structure, while the *cis* isomer features two well-separated peaks typical of the β-sheet structure in the polyQ core. The **AMPO** is colored purple, with polyQ in green and the charged termini in red for lysine and blue for aspartate.

Next, we used computational analysis to gain more insights into the structure of the more ordered fibrils formed by the *cis* isomer. The polyQ core structure itself (as detected by its ssNMR signature) was modeled based on previous studies and the molecular structure of the *cis* β-hairpin turn was optimized at the B3LYP/6-31G level of QM theory (Figure 7a–c).^9,42,43,46−48^ Indeed, the *cis* isomer was able to connect the antiparallel strands and form a β-turn, fulfilling the geometric requirements and not interfering with the H-bond formation of nearby Gln residues. This finding fits the ssNMR observations, which suggest that all or most of the ten Gln residues in the peptide must adopt the β-strand conformations typical of polyQ amyloids (Figures 7a,d, S15). For the *trans* configuration, few glutamines fold in β-sheets, and the others have a disordered structure. For this reason, in the ssNMR spectrum of the *trans* isomer, we cannot easily identify the characteristic peaks of form “a” and “b”, and instead, the peaks represent a combination of some residues adopting the “a” and “b” conformations, together with signals from more disordered residues (Figure S15). Furthermore, using the modeled structure, we measured the *cis***polyQ-AMPO** β-strand length being ∼3 nm, which matches the fibril width seen by TEM. It is worth noting that the supramolecular assembly of peptide monomers into fibrils permits multiple possible models proposed for *cis***polyQ-AMPO**. As illustrated in Figure 7b,c, the **AMPO** moiety in neighboring peptides could reside on the same side of the fiber (left) or it may alternate on both sides (right).

## Conclusions

The aim of this work was to design and test a new amino acid-based azobenzene that could be incorporated into polyQ (and other) amyloidogenic peptides to enable control over their aggregation with light. Knowing the structure of the amyloid fibril architecture and using computational analysis, we successfully designed a new amino acid-based azobenzene, **AMPO**, to better mimic the β-turn. We successfully placed the β-turn-mimicking **AMPO** structure within a polyQ peptide, generating a photoresponsive polyQ peptide. The UV–vis and the liquid-state NMR results confirmed the improved photochemical properties of both **AMPO** and the **polyQ-AMPO**. Both the *cis* and *trans* configurations formed aggregates, and their structure was studied with EM and ssNMR. EM proved their fibrillar shape, and ssNMR showed that the *cis***poly-AMPO** isomer successfully replicated the typical polyQ amyloid structure, whereas the *trans* cannot.

In conclusion, **polyQ-AMPO** successfully affords photochemical control over the monomeric and fibrillar structure of these amyloidogenic peptides. The **AMPO** photoswitches open up new avenues for the investigation of the role of β-harpin formation in the nucleation and propagation of amyloid formation in HD and related polyQ diseases. We also envision its use in the context of other processes, from amyloid formation in other diseases to controlling peptide-based materials based on β-hairpins.^22^ A deeper understanding of the molecular processes behind protein misfolding diseases is important for the design of aggregation-inhibiting and -modulating drugs and treatments.

## Supporting Information Available

Description of experimental procedures, photochemical characterization of samples in different solvents, solution and solid-state NMR data, and data on **AMPO** synthesis (PDF)
