# Supplementary material for: Photocontrol of the β-Hairpin Polypeptide Structure through an Optimized Azobenzene-Based Amino Acid Analogue
Source: J Am Chem Soc. 2024 Jan 16;146(3):2062–71. doi: 10.1021/jacs.3c11155 (PMC10811659; doi:10.1021/jacs.3c11155)
Supplement: Supplementary file 1 — ja3c11155_si_001.pdf [file ja3c11155_si_001.pdf]

Supporting Information for

Photocontrol of β-hairpin polypeptide structure through an optimized azobenzene-based amino acid analogue

*Raffaella Parlato[1] ‡, Jana Volarić[2] ‡, Alessia Lasorsa[1], Mahdi Bagherpoor Helabad[3], Piermichele Kobauri[2], Greeshma Jain[1], Markus Miettinen[4], Ben L. Feringa[2, *], Wiktor Szymanski[2,5, *] and Patrick C.A. van der Wel[1, *]*

[1] Zernike Institute for Advanced Materials, University of Groningen, Nijenborgh 4, 9747 AG, Groningen
[2] Stratingh Institute for Chemistry, University of Groningen, Nijenborgh 7, 9747 AG, Groningen
[3] Department of Theory and Bio-Systems, Max Planck Institute of Colloids and Interfaces, 14424 Potsdam, Germany
[4] Computational Biology Unit, Departments of Chemistry and Informatics, University of Bergen, 5020 Bergen, Norway
[5] Medical Imaging Center, University Medical Center Groningen, Hanzeplein 1, 9713 GZ, Groningen

1. Experimental section 3
1.1 General information 3
1.2 Design of AMPO 3
1.3 Chemical synthesis 4
1.4 Solubilization protocol for AMPO-PolyQ 6
1.5 UV-vis spectroscopy analysis 6
1.6 Solution NMR sample preparation 7
1.7 Solid-state NMR spectroscopy: 7
1.8 Transmission electron microscopy: 9
1.9 Computational analysis: 9
2. Supplementary Images 10
3. Analytical data of all synthesized compounds 29
4. References cited in the Supporting Information 36

1. Experimental section

1.1 General information

All chemicals for synthesis were obtained from commercial sources and used as received unless stated otherwise. Technical grade solvents were used for extraction and chromatography. Thin Layer Chromatography (TLC) was performed using commercial Kiesegel 60 F254 silica gel plates with fluorescence-indicator UV254 (Merck, TLC silica gel 60 F254). For detection of components, UV light at λ=254 nm or λ=365 nm was used. Alternatively, oxidative staining was performed using a basic solution of potassium permanganate in water or aqueous cerium phosphomolybdic acid solution (Seebach's stain). Merck silica gel 60 (230–400 mesh ASTM) was used in normal phase flash chromatography. Büchi Reveleris® X2 automatic column was used with Büchi EcoFlex silica columns (4 - 40 g, 40−63 µM, 60 Å). Liquid-state NMR spectra were obtained using Agilent Technologies 400-MR (400/54 Premium Shielded) (^{1}H: 400 MHz, ^{13}C: 101 MHz, ^{19}F: 61 MHz) and Bruker Innova (^{1}H: 600 MHz, ^{13}C: 151 MHz) spectrometers at room temperature (22-24 °C). Chemical shift values (δ) in the liquid-state NMR analysis are reported in parts per million (ppm) with the solvent resonance as the internal standard (TMS: δ 0.00 for ^{1}H, $CDCl_3$: δ 7.26 for ^{1}H, δ 77.16 for ^{13}C; DMSO: δ 2.50 for ^{1}H, δ 39.52 for ^{13}C; CD_3OD: δ 3.31 for ^{1}H, δ 49.0 for ^{13}C; D_2O: δ 4.79 for ^{1}H). The following abbreviations are used to indicate signal multiplicity: s (singlet), d (doublet), t (triplet), q (quartet), m (multiplet), brs (broad signal) or dd (double doublet). Exact mass spectra were recorded on an LTQ Orbitrap XL (ESI+, ESI- and APCI). All reactions requiring an inert atmosphere were carried out under a nitrogen atmosphere using oven dried glassware and standard Schlenk techniques. Dichloromethane (DCM) was used from a solvent purification system using an MBraun SPS-800 column. Melting points were determined using Stuart analogue capillary melting point SMP11 apparatus. All errors are given as standard deviations.

1.2 Design of AMPO

The conformational search on both photoisomers of the azobenzene-based amino acids **AMPB**, **AMPP** and **AMPO** was carried out with MacroModel with no constraints (Maestro ver 12.4, Schrödinger Release 2020-2: Maestro, Schrödinger, LLC, New York, NY, 2020). OPLS3e force field was used to perform an enhanced sampling, with the following parameters: method = TNCG, 50000 max iterations, convergence threshold 0.05, 62.8 kJ/mol window, elimination of redundant conformers with max atom deviation of 0.5 Å.

1.3 Chemical synthesis

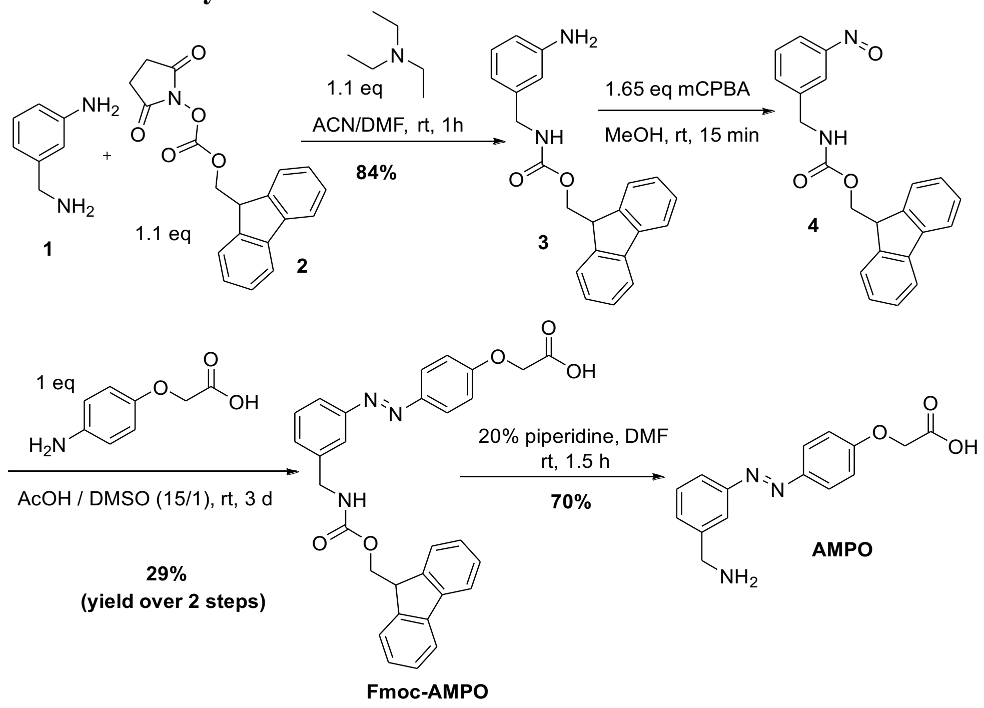

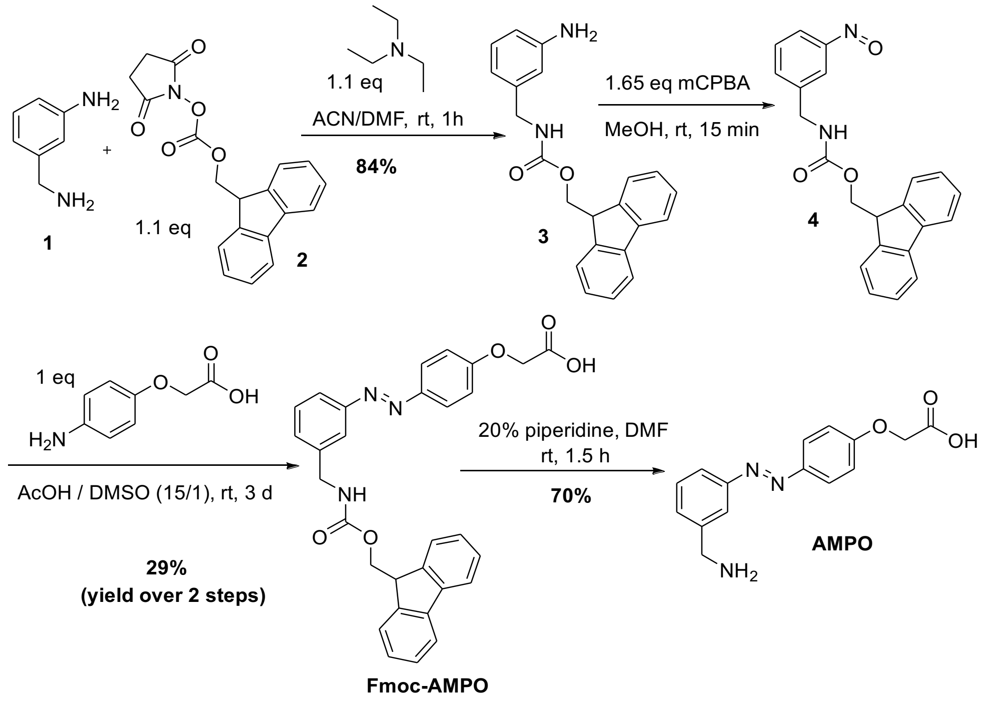

Scheme S1. Synthesis of **Fmoc-AMPO** and **AMPO**.

(9H-Fluoren-9-yl)methyl (3-aminobenzyl)carbamate (3)[1,2]

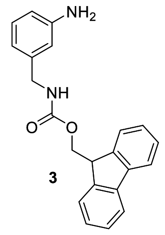

3-Aminobenzylamine 1 (2.0 g, 16 mmol) was dissolved in a mixture of acetonitrile (19 mL) and DMF (1.6 mL). Triethylamine (1.1 eq, 0.73 mL, 18 mmol) was added via a syringe. A solution of Fmoc-OSu (**2**) (1.1 eq, 6.0 g, 18 mmol in 40 mL of acetonitrile) was added drop-wise via a dripping funnel over 60 min. The formed white precipitate was filtered, washed with water and diethyl ether. The product was obtained as a white powder (4.78 g, 85% yield).

Mp. 127-130 °C. (lit. 142-143 °C);[1,2] ^{1}H NMR (400 MHz, DMSO-d_6) δ 7.89 (d, J = 7.4 Hz, 2H), 7.79 – 7.72 (m, 1H), 7.71 (d, J = 7.4 Hz, 2H), 7.42 (dd, J=7.42 Hz, 2H), 7.33 (t, J=7.4 Hz, 2H), 6.94 (t, J = 7.7 Hz, 1H), 6.48 – 6.35 (m, 3H), 5.00 (s, 2H), 4.30 (d, J = 7.0 Hz, 2H), 4.22 (t, J = 7.0 Hz, 1H), 4.04 (d, J = 6.2 Hz, 2H). The spectroscopic data is in accordance with the literature.[1,2]

(*E*)-2-(4-((3-(((((9H-Fluoren-9-yl)methoxy)carbonyl)amino)methyl)phenyl)diazenyl)phenoxy) acetic acid (Fmoc-AMPO)

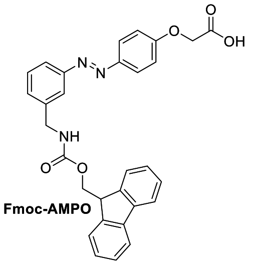

Aniline **3** (3.0 g, 8.6 mmol) and *m*CPBA (2.2 eq, 19 mmol, 75% purity) were dissolved in 250 mL of DCM and the mixture stirred for 15 min at room temperature. The green solution was washed four times with sat. aq $NaHCO_3$ (300 mL), dried over $MgSO_4$ and the solvent was evaporated to obtain **4** as a green solid that was immediately used in the next step. The crude nitroso product **4** and 2-(4-aminophenoxy)acetic acid (1.0 eq, 1.4 g, 8.3 mmol) were dissolved in glacial AcOH (70 mL) and DMSO (5 mL) in a 250 mL flask. The dark reaction mixture was left to stir at rt for 4 d. Subsequently, the resulting mixture was diluted with DCM (500 mL) and the solvent mixture (acetic acid and DMSO) was removed by nine washes with water (400 mL). The organic layer was dried over $MgSO_4$, and the solvent was evaporated. The product was purified by flash chromatography on silica gel with a solvent gradient from pure DCM to 20% MeOH in DCM to provide 1.2 g of product **5** as an orange solid (29 % yield over two steps).

Mp. 176-178 °C. ^{1}H NMR (400 MHz, DMSO-d_6) δ 8.00 (t, J = 6.2 Hz, 1H), 7.88 (d, J = 7.5 Hz, 2H), 7.82 (d, J = 8.6 Hz, 2H), 7.73 – 7.68 (m, 4H), 7.49 (t, J = 7.7 Hz, 1H), 7.43 – 7.28 (m, 5H), 7.01 (d, J = 8.6 Hz, 2H), 4.42 (s, 2H), 4.35 (d, J = 6.9 Hz, 2H), 4.29 (d, J = 5.9 Hz, 2H), 4.25 (s, 1H). ^{13}C NMR (600 MHz, DMSO-d_6) δ 161.8, 156.9, 152.5, 146.5, 144.3, 141.7, 141.2, 129.8, 129.7, 128.1, 127.5, 125.6, 124.8, 121.6, 120.8, 120.6, 115.6, 66.9, 65.9, 47.2, 44.0. HRMS (ESI+) calc for $C_{30}H25N_3O_5Na$: 530.1686, found 530.1679.

(*E*)-2-(4-((3-(Aminomethyl)phenyl)diazenyl)phenoxy)acetic acid (AMPO)

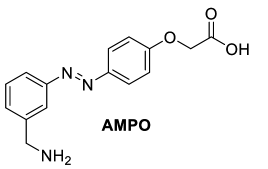

Fmoc-AMPO (100 mg, 0.195 mmol) was dissolved in 10 mL of 20% piperidine in DMF and left to stir at room temperature for 1.5 h under a nitrogen atmosphere. The product was precipitated by adding excess cold ether. Subsequently, the crude was washed with ether and centrifuged five times to obtain 38.9 mg of product 6 as a yellow solid (70% yield).

Mp. Decomp >246 °C. ^{1}H NMR (400 MHz, CD_3OD, a drop of TFA) δ 7.99 (d, J = 1.9 Hz, 1H), 7.96 – 7.90 (m, 3H), 7.69 – 7.53 (m, 2H), 7.11 (d, J = 9.0 Hz, 2H), 4.79 (s, 2H), 4.23 (s, 2H).^{13}C NMR (101 MHz CD_3OD, drop TFA) δ 172.2, 162.4, 160.5, 160.1, 154.5, 148.6, 131.9, 131.2, 125.9, 124.6, 123.6, 116.2, 66.0, 44.1. HRMS (ESI+) calc for $C_{15}H_{15}N_3O_3H$: 286.1186, found 286.1186.

PolyQ-AMPO (KKQQQQ-**AMPO**-QQQQQDD)
The **polyQ-AMPO** peptide, featuring 10 glutamines, two lysines at the *N*-terminus and two aspartates at the *C*-terminus, was synthesized by SPPS by Vivitide (Gardner, MA 01440, USA) using **AMPO** as a precursor starting material. The peptide was purified to 90-95% purity and the quality control was performed by High Performance Liquid Cromatography (HPLC).

1.4 Solubilization protocol for polyQ-AMPO

For solution-state analysis the peptide was treated following the disaggregation protocol used by Doran and coworkers, which is designed to remove or disaggregate traces of pre-aggregated peptide that can act as aggregation nuclei/catalysts.[3,4] The **polyQ-AMPO** peptide was dissolved in 1 mL of TFA for each mg of peptide and sonicated at room temperature for 1 minute. Next, TFA was carefully evaporated using a dry nitrogen gas stream, leaving a peptide film on the wall of the vial. Subsequently, the peptide was dissolved in 500 µL HFIP for each mg of peptide, incubated at 37 °C for 30 minutes and the solvent evaporated with a gentle nitrogen gas stream. The peptide was dissolved one more time in 1 mL of HFIP for each mg of peptide, which was evaporated with a nitrogen gas stream. Lastly, the sample was put under vacuum overnight. The obtained dry peptide film was dissolved in relevant solvents as indicated below. One exception to this disaggregation treatment were the experiments reported in figure S9 (page S17), where the lyophilized peptide was dissolved directly in 9% MeOH and water, without the disaggregation protocol.

1.5 UV-vis spectroscopy analysis

Spectroscopic measurements were made in Uvasol® grade solvents using a quartz cuvette (path length 10.0 mm). UV-Vis measurements were performed on an Agilent 8453 UV-Visible absorption spectrophotometer. UV-Vis irradiation experiments were carried out using a custom-built (Prizmatix/Mountain Photonics) multi-wavelength fiber coupled LED-system (FC6-LED-WL) with LED lights (365A and 445B), 470 nm LED light source (3x 470 nm, LED Nichia NCSB219B-V1, Sahlmann Photochemical Solutions), handheld UV lamp (Spectroline® 312 and 365 nm lamp with Long Life filter) and 365 nm LED (Thorlabs M365F1). The temperature was controlled with a Quantum Northwest TC1 temperature controller. The data was processed using Agilent UV-Vis ChemStation B.02.01 SP1, Spectragryph 1.2, OriginPro 2016 and all images were assembled in Adobe Illustrator.

The quantum yield of **Fmoc-AMPO** in DMSO and MeOH; **AMPO** in MeOH and TFA, K_2CO_3 and water and TFA; and **polyQ-AMPO** in 9% of MeOH, was measured as follows: triplicates of a known volume of the thermally adapted solution of **AMPO** and **polyQ-AMPO** in the solvents were irradiated with a custom-made irradiation set up (365 nm lamp) and the absorbance change in time was recorded.

The photon flux of the specific lamp was previously determined as described in reference.[5,6] Isomerization of *trans* **AMPO** to *cis* **AMPO** is a first order process. Therefore, the ln value of the absorbance change can be plotted against time giving a linear correlation from which slope the rate of reaction can be determined. This was done for the isomerization of thermally adapted **AMPO** and the initial values of the ln(A)-time curve where a linear correlation is observed were used to determine the rate of reaction k. Using the mean value of the three determined k_{fit} values, the quantum yield was calculated using equation:

$$\Phi_{(t\rightarrow c)} = \frac{(k_{fit}*c_0*N_A*V)}{\{I*[1-10^{(-A_0)}]\}} \quad \text{(Eq 1)}$$

where $\Phi_{(t\rightarrow c)}$ is the quantum yield of the observed process, k_{fit} is the determined rate constant, c_0 the initial concentration of the photoswitch, N_A is the Avogadro's constant, V volume of the irradiated sample, I is the photon flux of the lamp set up and A_0 is the initial absorbance at the irradiation λ.

1.6 Solution NMR sample preparation

Solution NMR samples were prepared at a concentration of 1 mM at room temperature. The liquid-state NMR spectra were obtained using Agilent Technologies 400-MR (400/54 Premium Shielded) (400 MHz) and Bruker Innova (^{1}H: 600 MHz, ^{13}C: 151 MHz) spectrometers at room temperature (22-24 °C). Chemical shift values (δ) are reported in parts per million (ppm) with the solvent resonance as the internal standard (TMS: δ 0.00 for ^{1}H, $CDCl_3$: δ 7.26 for ^{1}H, δ 77.16 for ^{13}C; DMSO: δ 2.05 for ^{1}H, δ 39.52 for ^{13}C, CD_3OD: δ 3.31 for ^{1}H, δ 49.0 for ^{13}C).

Solution NMR spectroscopy was used to estimate the Photostationary State Distribution (PSD) of irradiated *cis* **AMPO** at 365nm (PSD_{365nm}) and of irradiated *trans* **AMPO** at 470nm (PSD_{470nm}). The thermally equilibrated sample was measured in solution NMR and then irradiated *ex-situ* at 365nmm with a handheld UV lamp (Spectroline® 312 and 365 nm lamp with Long Life filter) to convert to the *cis* configuration. After, the sample in *cis* configuration (PSD_{365nm}) was measured again in solution NMR and irradiated *ex-situ* one more time at 470nm with a LED light source (3x 470 nm, LED Nichia NCSB219B-V1, Sahlmann Photochemical Solutions) to change it to PSD_{470nm}. Subsequentially, the three NMR spectra were compared and peaks appearing only in the *cis* or *trans* spectrum were selected and integrated. Considering the *trans* as the thermally equilibrated sample (99% of *trans* isomer) and integrating the selected peaks was possible to estimate the PSD of *cis* **AMPO** at 365nm and the *trans* **AMPO** at 470nm.

1.7 Solid-state NMR spectroscopy

According to the disaggregation protocol mentioned above, 6 mg and 3 mg of **polyQ-AMPO** were prepared to create *trans* and *cis* samples as follows. Both samples were dissolved in TFA (1 mL for 1 mg) with a peptide concentration of 0.5 mM and sonicated for 1 minute at r.t. Then the TFA was evaporated under a gentle nitrogen stream. The film of both samples was redissolved in HFIP (250µL for 1mg of peptide) for 30 minutes at 37°C and subsequentially the HFIP was evaporated with nitrogen

stream. Afterward, the samples were dissolved in HFIP (500µL for 1 mg of peptide) one more time. The *cis* sample was irradiated at 365nm with a handheld UV lamp (Spectroline® 312 and 365 nm lamp with Long Life filter). The HFIP was evaporated in both *cis* and *trans* samples and placed under vacuum overnight. Finally, both samples were dissolved in PBS (137mM Sodium Chloride, 2.7mM Potassium Chloride, 10 mM Sodium Phosphate Dibasic, 1.8 mM Potassium Phosphate Monobasic) (1X, pH 7, r.t.) at the peptide concentration of 1mM and allowed to aggregate for 96 hours. The end result of this process was the presence of yellow aggregates easily visible by eye.

The thus-prepared **polyQ-AMPO** *cis* and *trans* aggregates were packed into 3.2 mm zirconia regular wall MAS rotors (Bruker Biospin, Billerica, MA) by pelleting the hydrated suspension. This sedimentation process was performed using an ultracentrifugal packing device under centrifugation at ~130,000g in a Beckman Coulter Optima LE-80K ultracentrifuge equipped with a SW-32 Ti rotor.[7] Excess buffer was removed before sealing the cap of the rotors. The amount of *trans* **polyQ-AMPO** and *cis* **polyQ-AMPO** in each MAS rotor was 6mg and 3mg, respectively, representing the estimated dry weight equivalents. Note that these ssNMR samples were studied in a hydrated and unfrozen state. All spectra were acquired on a Bruker Avance 600 MHz spectrometer, equipped with a 3.2 mm MAS probe with an HCN Efree coil (Bruker Biospin). All the experiments were recorded with a spinning rate of 13kHz and at 275K.

^{1}H and ^{13}C ssNMR chemical shifts were referenced to aqueous DSS using the indirect method, by measuring adamantane ^{13}C signals.[8] The 1D ^{13}C insensitive nuclei enhanced by polarization transfer (INEPT) and cross polarization (CP) MAS spectra were acquired under the following conditions: ^{1}H 90° pulse was set to 2.5 µs corresponding to a rf power of 100 kHz; the CP step was performed with contact time (CT) of 1 ms using a 70−100 % ramped-amplitude (RAMP) shape on the ^{1}H channel and using a 50 kHz square shape pulse on the ^{13}C channel; recycle delay was 3 s. During acquisition, a TPPM (two-pulse phase-modulated) decoupling scheme was employed using a pulse length for the basic decoupling unit of 5.8 µs at rf field strength of 83 kHz.[9] The 1D ^{13}C CP and INEPT experiments were recorded with 2048 and 1024 scans per spectrum respectively (total experimental time of 103 minutes for CP experiments and 51.5 minutes for INEPT experiment). The 2D ^{1}H-^{13}C LG-CP HETCOR spectra were recorded on the **polyQ-AMPO** and on U-13C,15N-Δ-N15-Q44-HttEx1 fibrils.[10] The HETCOR spectra were acquired using Frequency-Switched Lee-Goldburg (FSLG) ^{1}H homonuclear decoupling during the indirect dimension (t_1).[11,12] LG-CP was employed to achieve ^{1}H to ^{13}C magnetization transfer, using CT of 100 µs and 350 µs, ^{13}C rf amplitude of ~50 kHz and 4 s recycle delay. The number of t_1 points was 104, with 128 scans recorded for each. A ^{1}H rf field strength of 100 kHz and symmetric LG offsets of 70711 Hz and -70711 Hz were used for FSLG decoupling employing a LG pulse length of 8.17 µs. Quadrature detection in t_1 was achieved by the States-TPPI method. 1D ^{13}C ssNMR spectra were processed using TopSpin 4.07, while 2D ^{13}C-^{1}H HETCOR spectra were processed using NMRpipe, [13] and images exported from CcpNmr software suite v.2.5. Spectra carried out on the polyQ reference sample have been processed by applying an adjustable sine bell window function, and spectra recorded on the **polyQ-AMPO** peptides have been processed by applying a squared sine bell window function. Digital resolution has been increased by applying the zero-filling function, to a final number of points

four times the actual size along the indirect dimension in case of the **polyQ-AMPO** spectra and two times the actual size in case of the polyQ reference spectra.

1.8 Transmission electron microscopy

An aliquot from the *cis* and *trans* ssNMR samples preparation was used to perform Transmission Electron Microscopy (TEM). TEM was performed on mature *cis* and *trans* **polyQ-AMPO** protein fibrils which were allowed to aggregate at room temperature for 96 hours in PBS at the concentration of 1mM at pH 7. For the imaging studies, 200 mesh copper grids (SKU FCF200-Cu-50, Electron Microscopy Sciences, Hatfield, PA) with a carbon support film were utilized. The grids were glow-discharge for 0.5-1 min and then, a drop of the *cis* and *trans* fibrils samples was placed on it. Both samples were washed with MiliQ and the excess was removed by blotting. The uranyl 2% (wt/vol) acetate was used as negative staining and it was applied immediately after for 0.5-1 min. Afterwards, the excess of stain was removed and the grids were left to air dry. To record the EM images, a Philips CM120 electron microscope operating at 120kV with a slow scan CCD camera (Gatan) was used. For each fiber, the widths were measured three times and each measurement spanned the length of the negative stained area of the fibril with similar contrast. To estimate the width calculation for each sample, the ImageJ tool was used.[14]

1.9 Computational analysis

The **polyQ-AMPO** monomer model was prepared based on a previous computational model of interdigitated antiparallel β-sheets of polyQ.[15,16] The *cis* configuration of the azobenzene molecule was fitted between two neighboring Q_5 monomers in the β-sheet as informed by the experimentals (Figure 7a), and the D_2 and K_2 ends added as extensions of the β-strands. The generated structural model of **polyQ-AMPO** was then optimized by Density Functional Theory calculations using B3LYP with 6-31G (d, p) level of Quantum Mechanical theory in the Gaussian 03 set of quantum chemistry codes.[17] Two possible (head-to-head and head-to-tail) dimer alignments of such *cis* monomers were optimized similarly.

2. Supplementary Images

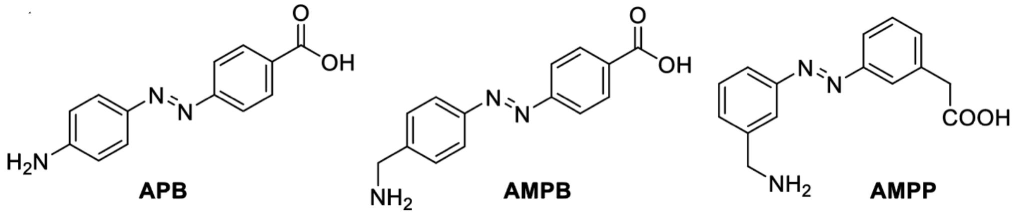

Figure S1. Azobenzene-based amino acids for introduction of the photoswitch into the backbone of the peptide.

Table S1. Photochemical properties of the evaluated azobenzenes

	**Compound**	**APB**	**AMPB**	**AMPP**	**AMPO**
Trans	**λ_{max} (nm)**	370[18]	332[19]	330[20]<br>322[21]	365
	**PSD**	-		80%(MeOH) [22]	90% (K_2CO_3)
	**QY** ***trans→cis***	0.2[18]	High[23]	-	-
Cis	**λ_{max} (nm)**	450[18]	429-435[19]	430[20]	470
	**PSD**	78%[18]	80% (360 nm)[23]<br><br>56% (AMPB-Zn finger) [19]<br>67 % (AMPB-AT-hook) [19]<br><br>75-80% (β-hairpin peptide, TFE/water=1/1)[24]<br><br>82:18 (366 nm, phosphate Buffer, pH 6.5)[25]	80% (MeOH)[22]<br>>65%[20]<br>84%[21]	65% (K_2CO_3)
	**QY** ***cis→trans***	0.5[18]	High[23]<br><br>0.22[24]<br>0.7 (Azopeptide)[24]<br><br>87% (430 nm, phosphate Buffer, pH 6.5) [25]	0.2 non-aggregated β-hairpin peptide[26]<br>0.02 aggregated[26]	0.71 (K_2CO_3)<br>0.13 (MeOH+H_20)
	$t_{1/2}$	10 min (DMSO, 22 °C) [18]<br>62 h (cyclic peptide)[18]	>50 h (AMPB-AT-hook) [19]<br>36h (AMPB-Sp1) [19]<br><br>~48 h (Azopeptide, 50 °C, EtOH)[24]	>2 weeks (20 mM acetate buffer at pH 3.8, 25 °C)[2]	>98h (K_2CO_3)

Table S2. ssNMR ^{13}C chemical shifts of assigned residues in **PolyQ-AMPO** *cis* and *trans* samples. The ^{13}C ssNMR chemical shifts were referenced to aqueous DSS using the indirect method, by measuring adamantane ^{13}C signals.[8] The maximum standard deviation of the ^{13}C chemical shifts is ± 0.3 ppm based on the systematic comparison, during the peak assignment process, of peak positions between the different spectra reported here. The peak position was determined using the peak maxima value.

		**C'**	**Cα**	**Cβ**	**Cγ**	**Cδ**	**Cε**
Trans	Gln 'a'	175.4	55.3	34.1[1]	34.1[1]	178.3	
	Gln 'b'	174.0	54.3	31.9	30.3	177.4	
	Lys						42.01
	Asp			42.0			
Cis	Gln 'a'	175.7	56.0	34.1[1]	34. 1[1]	178.6	
	Gln 'b'	174.1	54.2	31.9	30.3	177.5	
	Lys						42.0
	Asp			42.0			
U- ^{13}C, ^{15}N- Q_6- Q_{30} and Δ-N15-Q44-HttEx1 fibrils	Gln 'a'	175.9	56.1	34.2[1]	34.2[1]	178.4	
	Gln 'b'	173.9	53.9	31.7	30.6	177.6	

[1] Gln "a" Cβ and Cγ peaks of *cis*, *trans, Δ-N15-Q44-HttEx1 fibrils* and *U- ^{13}C, ^{15}N- Q_6- Q_{30}* overlap.

Table S3. ssNMR^{1}H chemical shifts of assigned residues in **polyQ-AMPO** *cis* and *trans* samples. The ^{1}H ssNMR chemical shifts were referenced to aqueous DSS using the indirect method, by measuring adamantane ^{13}C signals.[8] The maximum standard deviation of the ^{1}H chemical shifts is ± 0.3 ppm based on the systematic comparison, during the peak assignment process, of peak positions between the different spectra reported here. The peak position was determined using the peak maxima value.

		**Hα**	**Hβ**	**Hγ**
Trans	Gln 'a'	5.4	3.0	3.0
	Gln 'b'	5.5	3.1	2.9
	Lys			
	Asp			
Cis	Gln 'a'	5.5	2.9	2.9
	Gln 'b'	5.7	2.7	2.9
	Lys			
	Asp			
U- ^{13}C, ^{15}N- Q_6-Q_{30} and Δ-N15-Q44-HttEx1 fibrils	Gln 'a'	5.4	2.8	2.8
	Gln 'b'	5.7	2.7	2.6

Table S4. Experimental condition details of ssNMR experiments. Abbreviations: NS, number of scans; Temp., temperature; MAS, magic angle spinning rate; RD, recycle delay; TPPM, ^{1}H decoupling power during evolution and acquisition using the two-pulse phase modulation scheme.

Figures	Sample	Exp. Type	NS	Temp (K)	MAS (kHz)	RD (s)	TPPM (kHz)	Contact time (ms)	Evolution time (ms)
Fig. 5c (top), fig. S11a and S15 b	**polyQ-AMPO** *trans*	^{13}C CP	20480	275	13	3	83.3	1	NA
Fig. 5d (top), fig. S11b and fig. S15 a	**polyQ-AMPO** *cis*	^{13}C CP	30720	275	13	3	83.3	1	NA
Fig.1f, 5c, d (middle) and fig S15c	U- ^{13}C, ^{15}N- Q_6-Q_{30}	^{13}C CP	2048	275	13	3	83.3	1	NA
fig. S11c	**polyQ-AMPO** *trans*	^{13}C INEPT	1024	275	13	3	83.3	NA	NA
**Figure**	**Sample**	**Experiment**	**NS**	**Temp (K)**	**MAS (kHz)**	**RD (s)**	**TPPM (kHz)**	**Contact time (μs)**	**Evolution time (ms)**
S12 a	**polyQ-AMPO** *trans*	^{1}H-^{13}C CP	128	275	13	3	83.3	100	3.9
S12 c	Δ-N15-Q44-HttEx1 fibrils	^{1}H-^{13}C CP	128	275	13	3	83.3	100	3.9
S12 e	**polyQ-AMPO** *cis*	^{1}H-^{13}C CP	128	275	13	3	83.3	100	3.9
S12 b	**polyQ-AMPO** *trans*	^{1}H-^{13}C CP	128	275	13	3	83.3	350	3.9
S12 d	Δ-N15-Q44-HttEx1 fibrils	^{1}H-^{13}C CP	128	275	13	3	83.3	350	3.9

Figures	Sample	Exp. Type	NS	Temp (K)	MAS (kHz)	RD (s)	TPPM (kHz)	Contact time (ms)	Evolution time (ms)
S12 f	**polyQ-AMPO** *cis*	^{1}H-^{13}C CP	128	275	13	3	83.3	350	3.9

a

Peptide sequence	PDB name	Peptide’s origin
YVVFL	4ONK	Enkephalin Mutants
NFVFGT	6DIX	Human immunoglobulin
GAIIGL	3PZZ	Aβ Amyloid from Alzheimer
MVGGVVIA	2Y3L	Aβ Amyloid from Alzheimer
MVGGVV	2ONA	Aβ Amyloid from Alzheimer

b

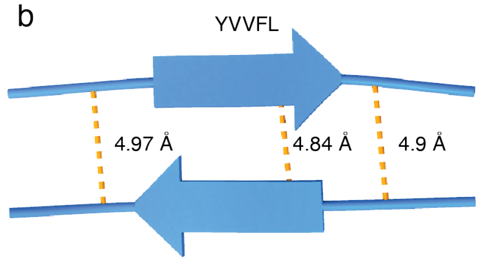

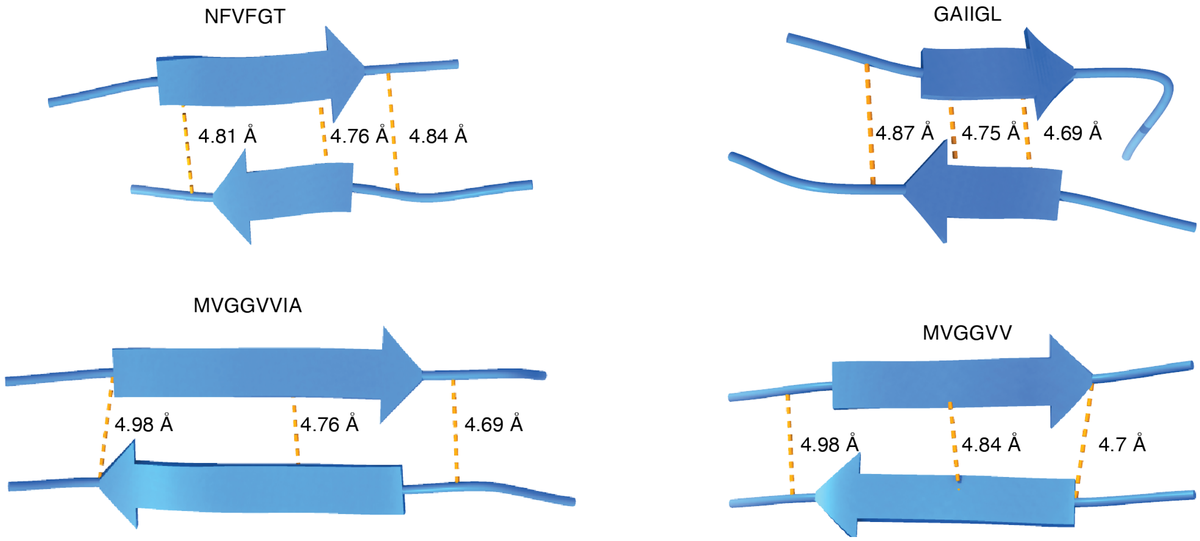

Figure S2. Antiparallel β-sheet amyloid model structures used for **AMPO** design analysis. In the design process of the new **AMPO** module, we aimed to improve the geometric incompatibilities of known constructs with antiparallel (β-hairpin) amyloid structures (see main text). Therefore, we examined known structures of a set of model antiparallel β-sheet amyloids. a) Table showing the amino acid sequence, the PDB file name, and parent amyloidogenic protein for the examined antiparallel amyloid-like architectures depicted in panel b. b) The structure of selected peptides analyzed using UCSF ChimeraX (version 1.6rc). The characteristic strand-to-strand distances for each structure are indicated, reflecting distances between atoms Carbon alpha and Nitrogen in the backbone.

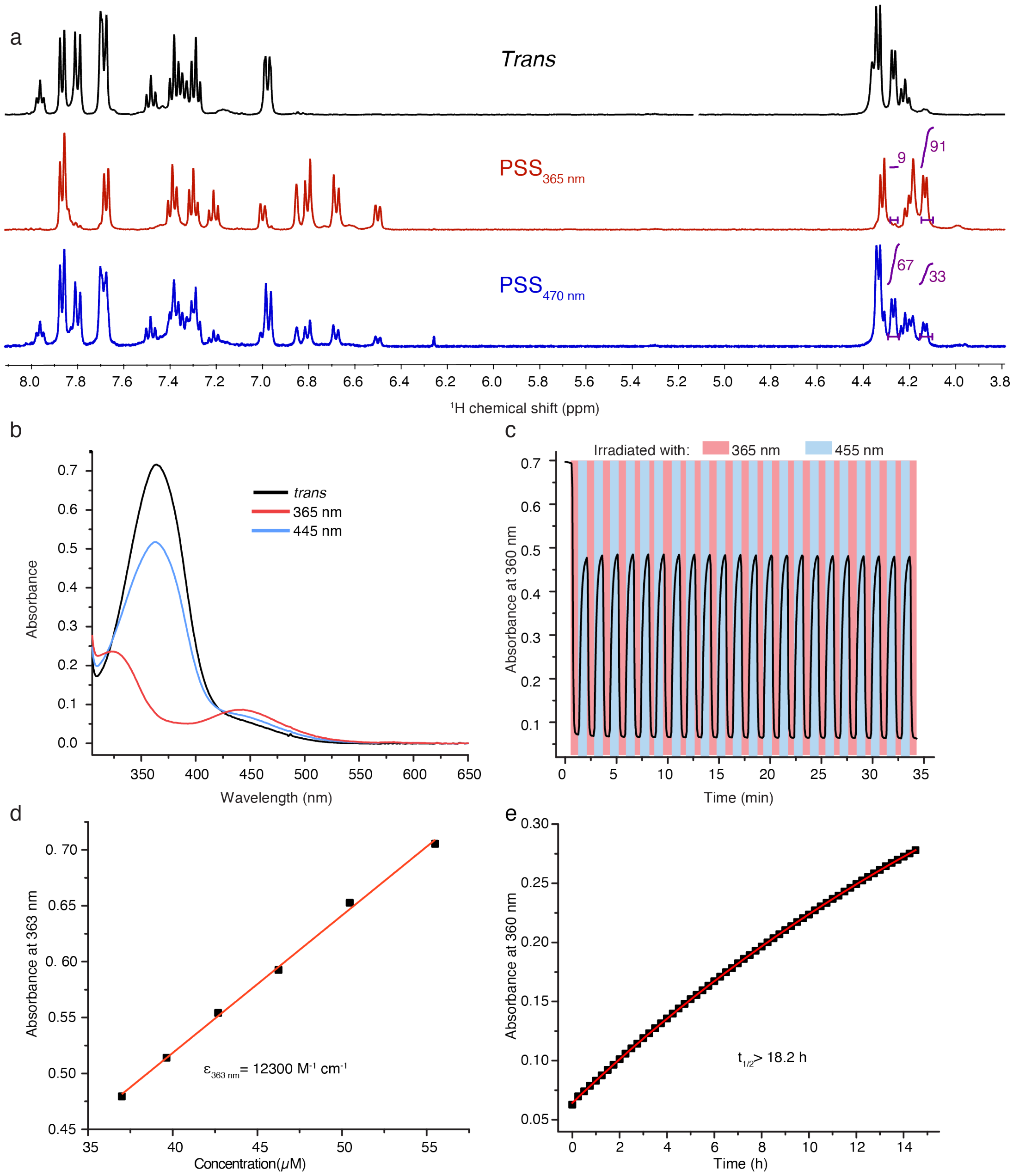

Figure S3. Photochemical properties of the **Fmoc-AMPO** in DMSO at 25 µM and 20 °C. a) ^{1}H NMR spectroscopy determination of PSS of **Fmoc-AMPO** in *trans* (black), Photostationary State (PSS) at 365 nm (red) and PSS at 455 nm (blue) in DMSO-d_6 at 1.0 mM concentration. The unique peaks in the *cis* and *trans* spectra were integrated to calculate the PSD. Considering the *trans* as the thermally equilibrate sample (99% of *trans* isomer) using peak integrating was possible to estimate the ratio between *trans* and *cis* isomer after light irradiation at 365nm and 470nm. b) Absorbance spectra of the *trans* (black), PSD at 365 nm (red) and PSD at 455 nm (light blue). c) Fatigue resistance test after irradiation with 365 (pink) and 455 nm (light-blue). The sample was switch between *trans* and *cis* configuration repetitively for 35 minutes. d) Determination of the molar absorptivity at the absorbance maximum at 363 nm. e) Half-life determination.

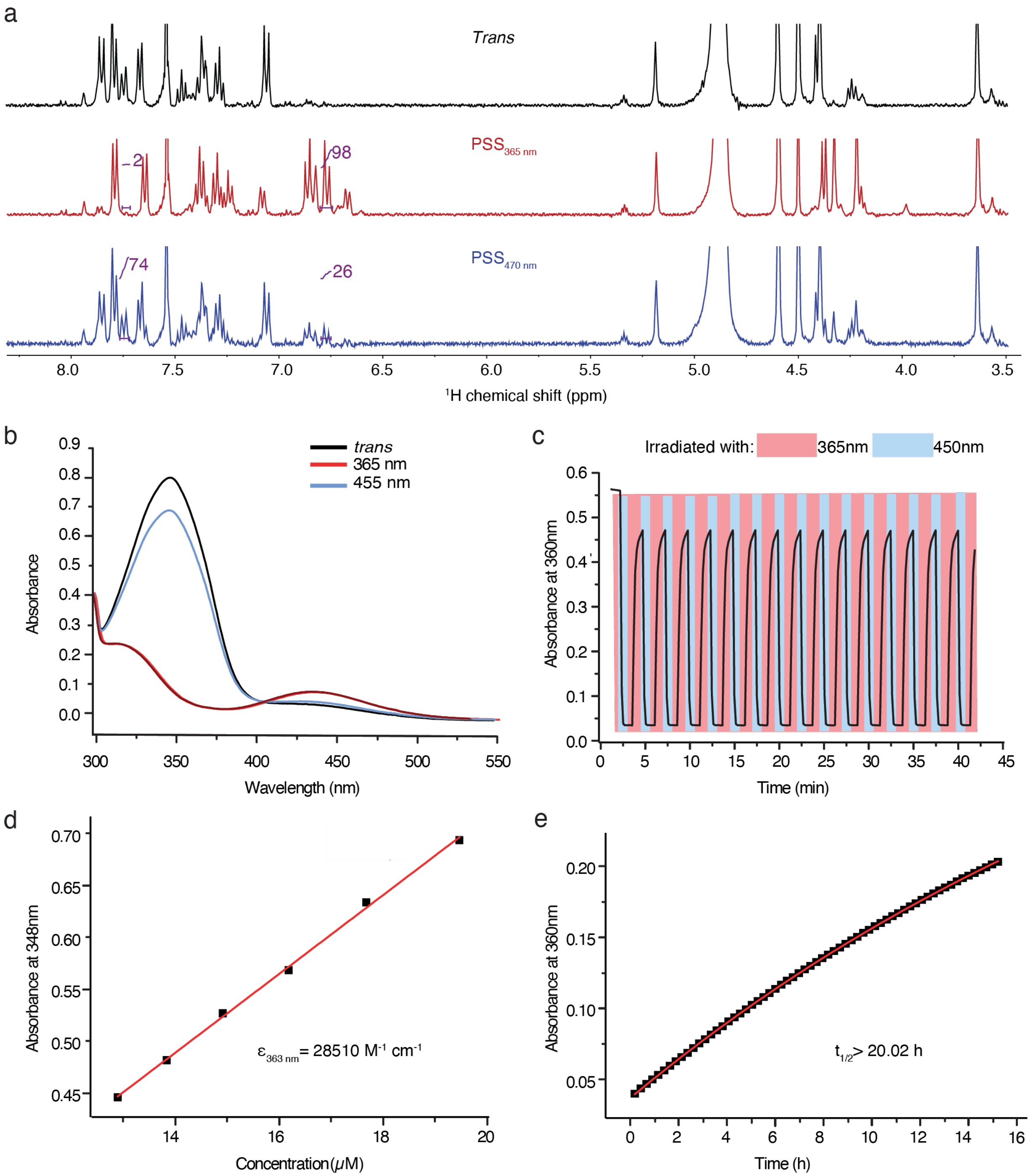

Figure S4. Photochemical properties of the **Fmoc-AMPO** in MeOH at 25 µM and 20 °C. a) ^{1}H NMR spectroscopy determination of PSS of **Fmoc-AMPO** in MeOD at 1.0 mM concentration. b) Absorbance spectra of the *trans* (black), PSD at 365 nm (red) and PSD at 455 nm (light-blue). c) Fatigue resistance test after irradiation with 365 (pink) and 455 nm (light-blue). d) Determination of the molar absorptivity at the absorbance maximum at 348 nm. e) Half-life determination.

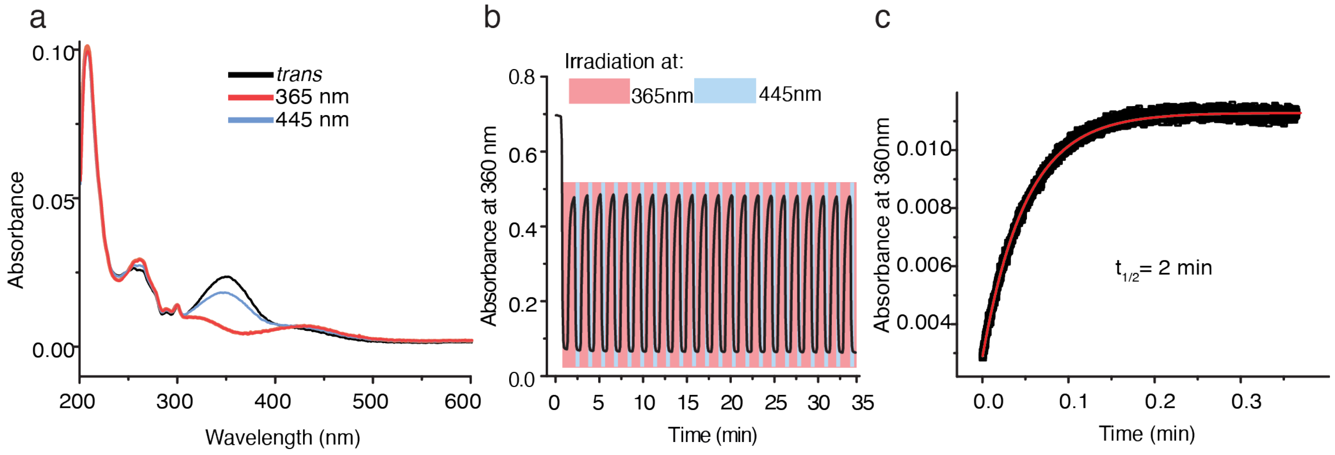

Figure S5. Photochemical properties of **AMPO** in 1.0 mM aqueous K_2CO_3 solution (pH 8) and 20 °C. a) Absorbance spectra of the *trans* (black), PSD at 365 nm (red) and PSD at 455 nm (light-blue). b) Fatigue resistance test after irradiation with 365 (pink) and 455 nm (light-blue). c) Half-life determination.

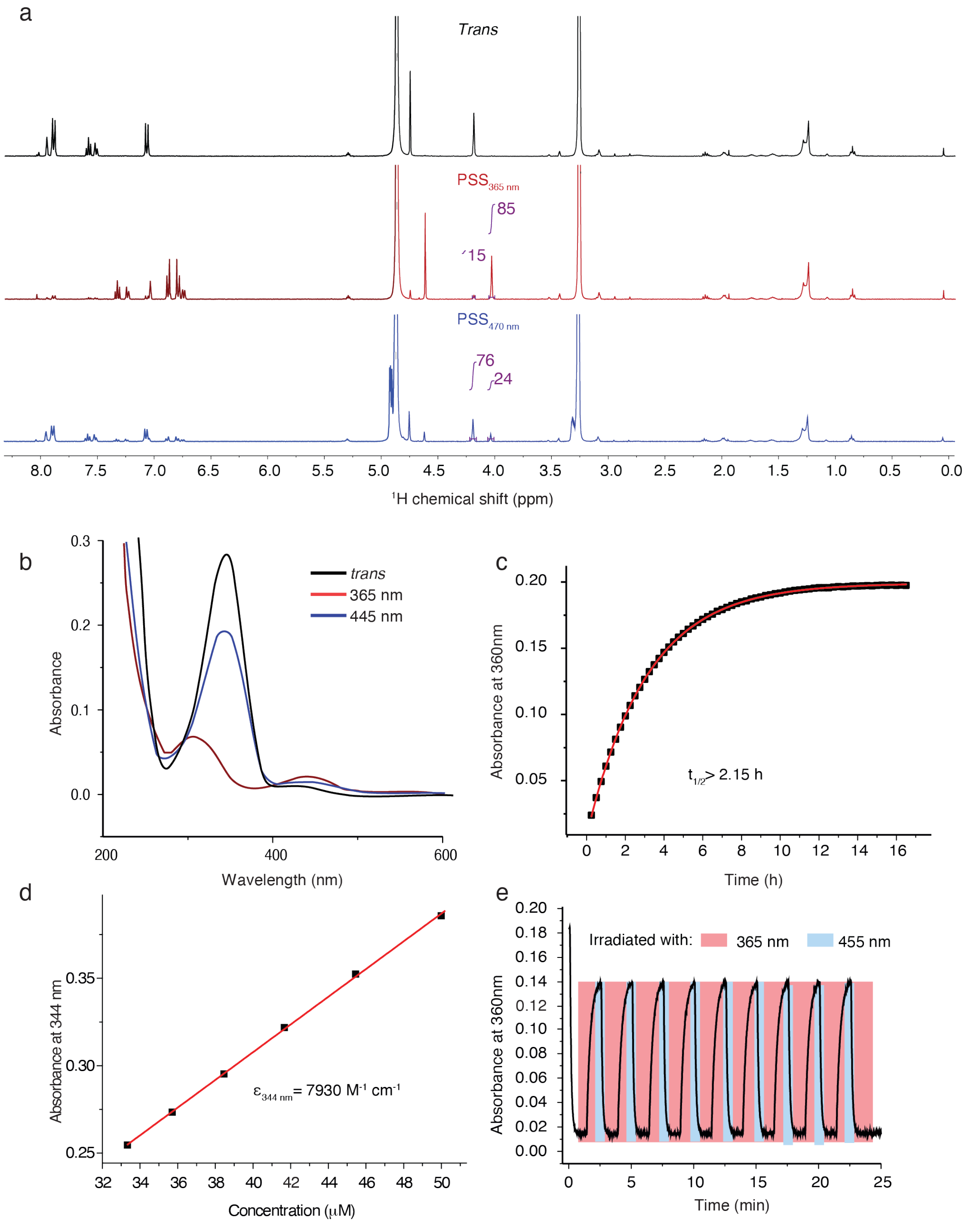

Figure S6. Photochemical properties of **AMPO** in MeOH with a drop of TFA (pH 2) at 25 μM and 20 °C. a) ^{1}H NMR spectroscopy determination of PSS of **AMPO** in in MeOD+TFA at 1.0 mM conc. b) Absorbance spectra of the *trans* (black), PSD at 365 nm (red) and PSD at 455 nm (light-blue). c) Half-life determination. d) Determination of the molar absorptivity at the absorbance maximum at 344 nm. e) Fatigue resistance test after irradiation with 365 and 455 nm.

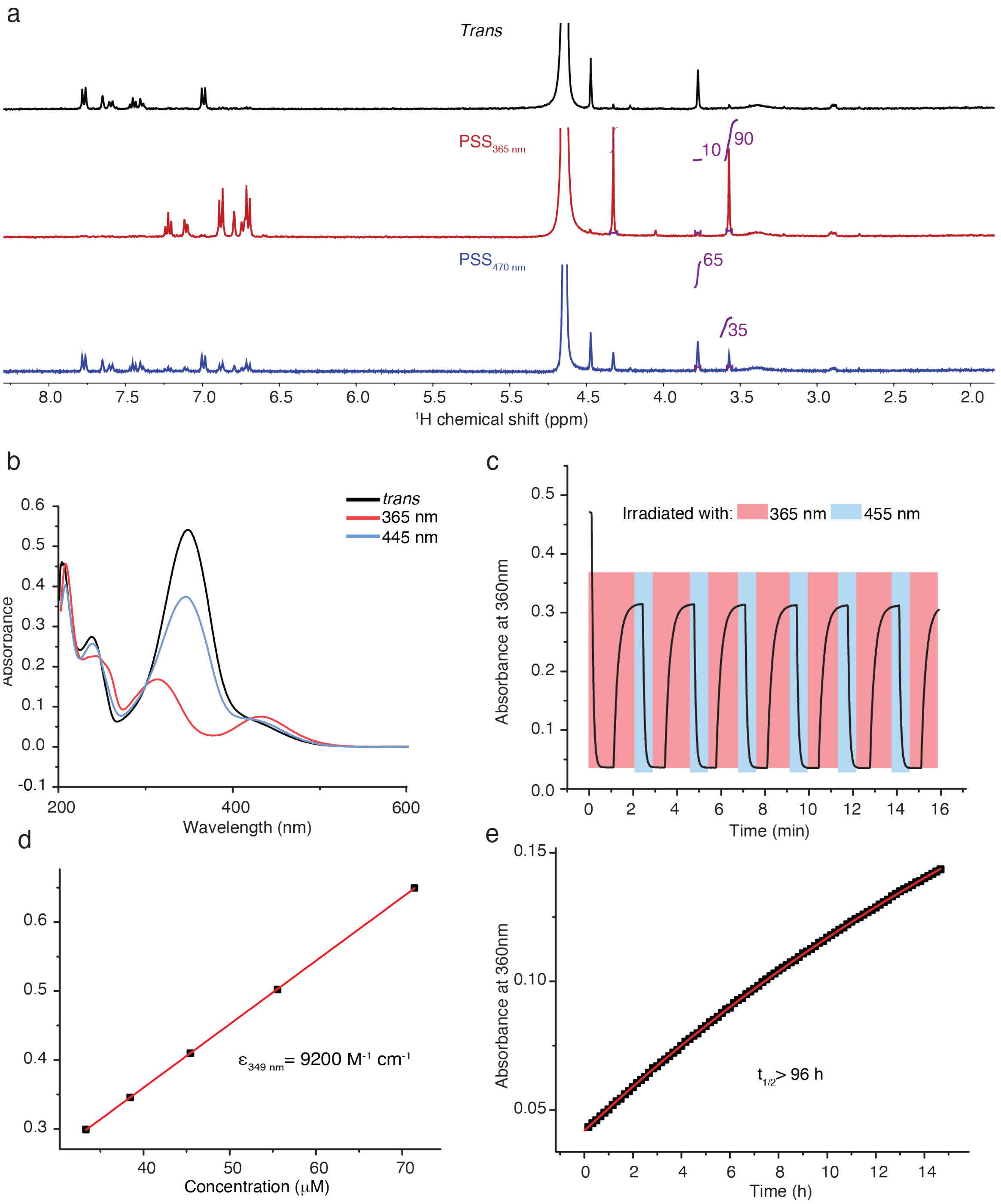

Figure S7. Photochemical properties of **AMPO** in 1 mM aqueous K_2CO_3 solution (pH 8) at 25 µM and 20 °C. a) ^{1}H NMR spectroscopy determination of PSS of **AMPO** in aqueous solution (D_2O) of K_2CO_3 at 1.0 mM conc. b) Absorbance spectra of the *trans* (black), PSD at 365 nm (red) and PSD at 455 nm (light blue). c) Fatigue resistance test after irradiation with 365 and 455 nm. d) Determination of the molar absorptivity at the absorbance maximum at 349 nm. e) Half-life determination.

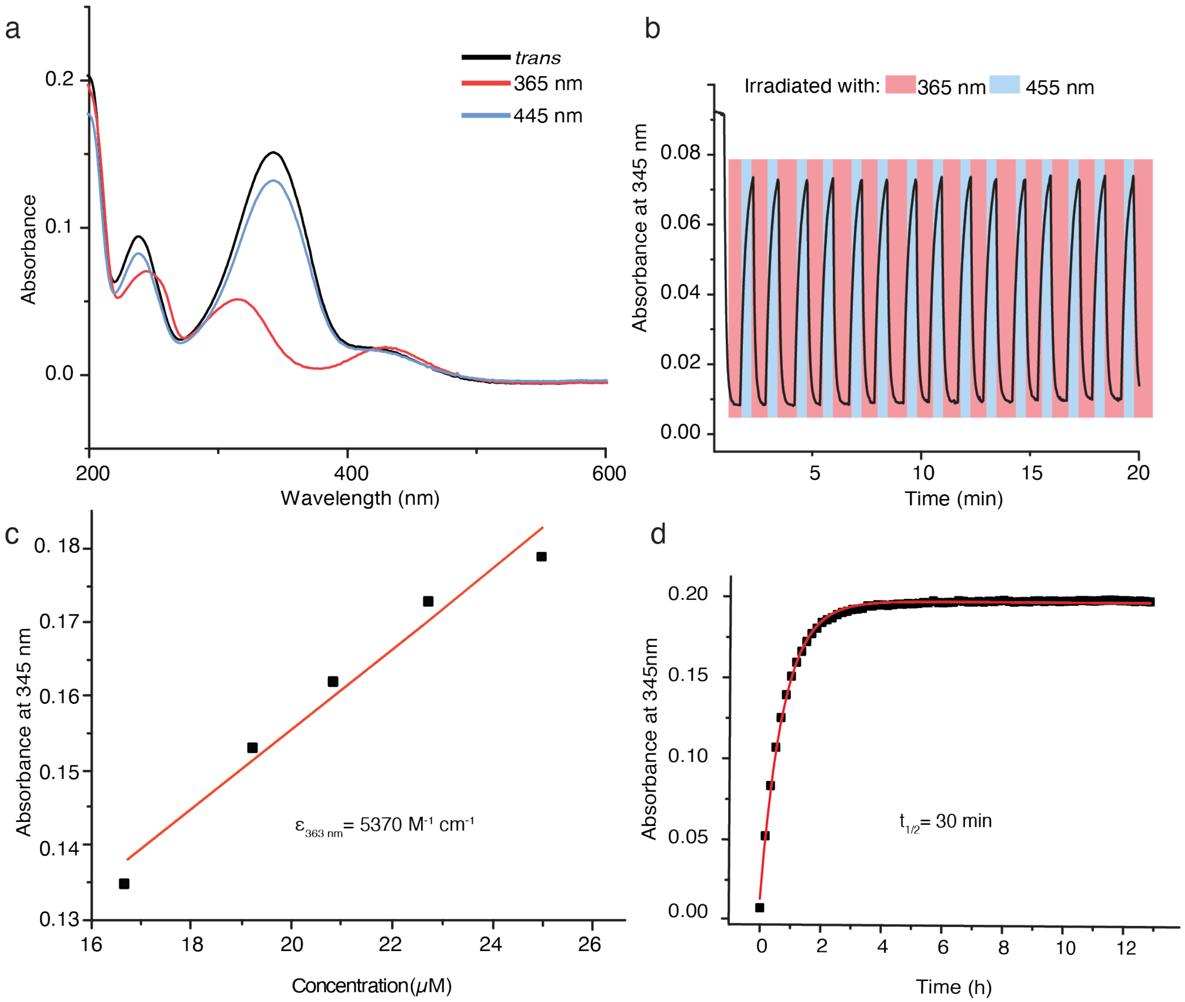

Figure S8. Photochemical properties of **AMPO** in water and a drop of TFA (pH 2) at 25 µM and 20 °C. a) Absorbance spectra of *trans* (black), PSD at 365 nm (red) and PSD at 455 nm (light blue). b) Fatigue resistance test after irradiation with 365 and 455 nm. c) Determination of the molar absorptivity at the absorbance maximum at 345 nm. d) Half-life determination.

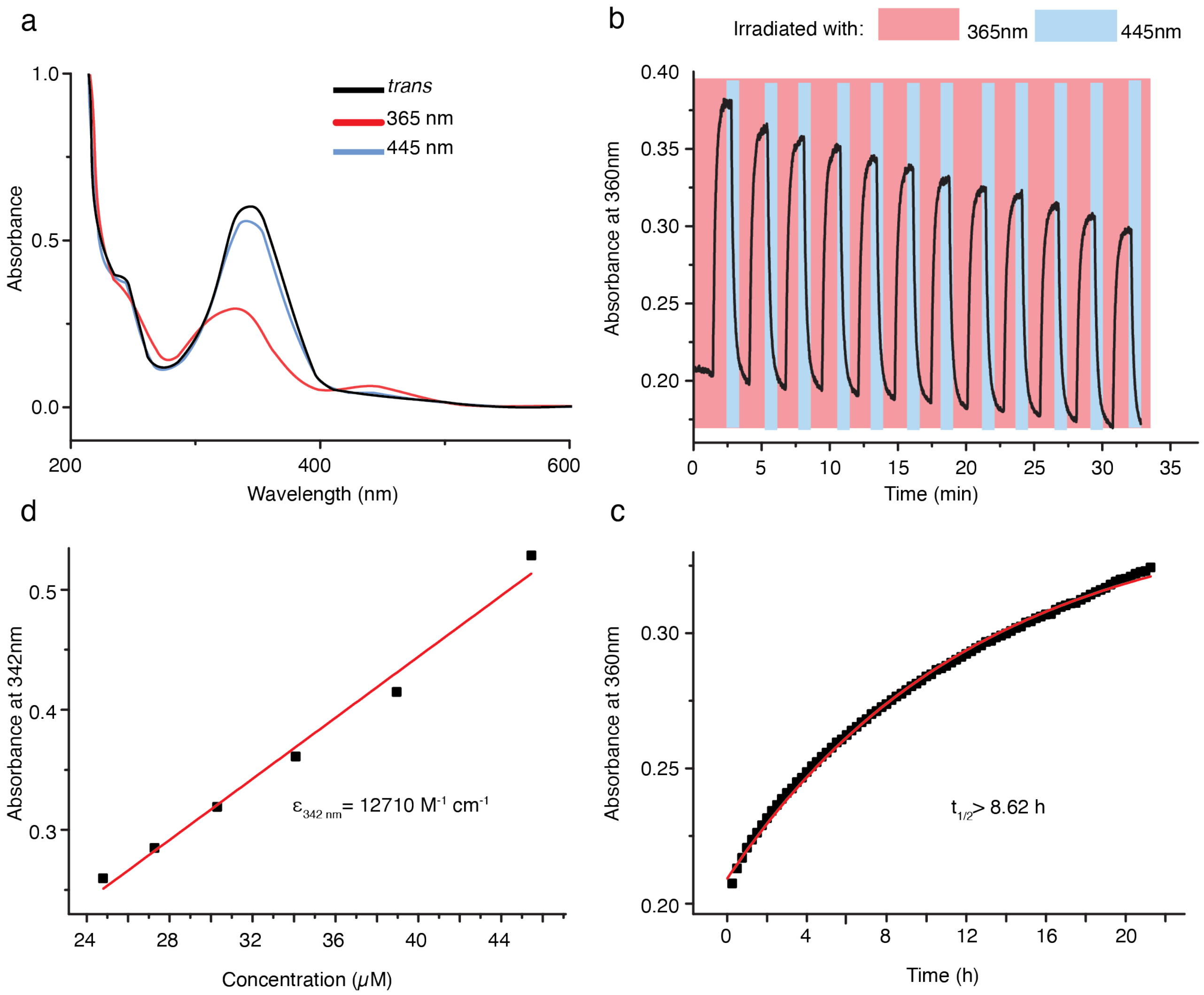

Figure S9. Photochemical properties of **polyQ-AMPO** before disaggregation protocol in 9% MeOH and water at 25 µM and 20 °C.[3,4] a) Absorbance spectra of *trans* (black), PSD at 365 nm (red) and PSD at 455 nm (light-blue). b) Fatigue resistance test after irradiation with 365 and 455 nm. c) Determination of the molar absorptivity at the absorbance maximum at 342 nm. d) Half-life determination.

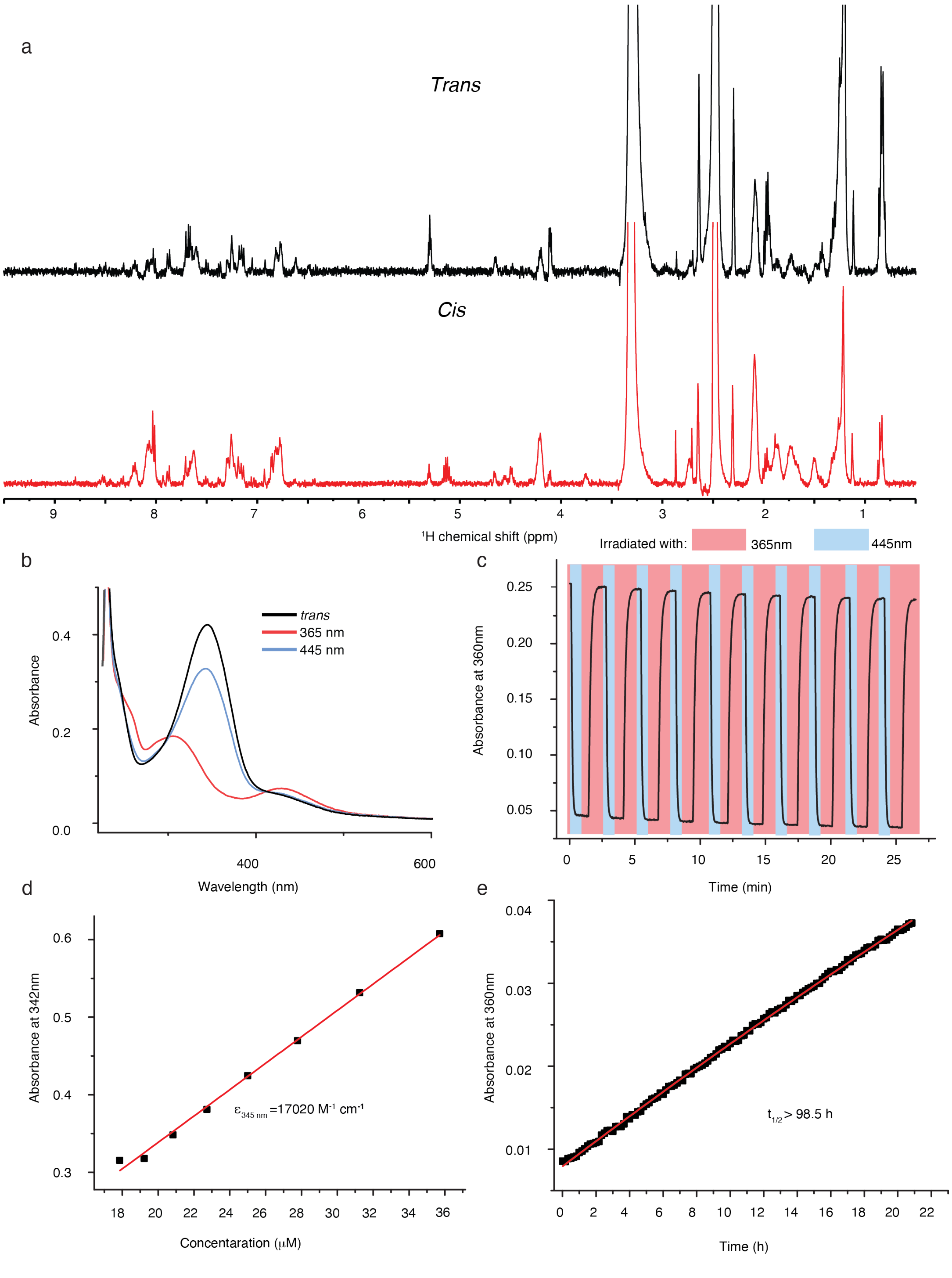

Figure S10. Photochemical properties of the **polyQ-AMPO** in PBS and 0.5% of DMSO at 25 μM and 20 °C. a) ^{1}H NMR spectroscopy of *trans* (black) and *cis* (red) configuration in DMSO after the disaggregation protocol.[3,4] b) Absorbance spectra of the *trans* (black), PSD at 365 nm (red) and PSD at 455 nm (light-blue). c) Fatigue resistance test after irradiation with 365 and 455 nm. d) Determination of the molar absorptivity at the absorbance maximum at 349 nm. e) Half-life determination.

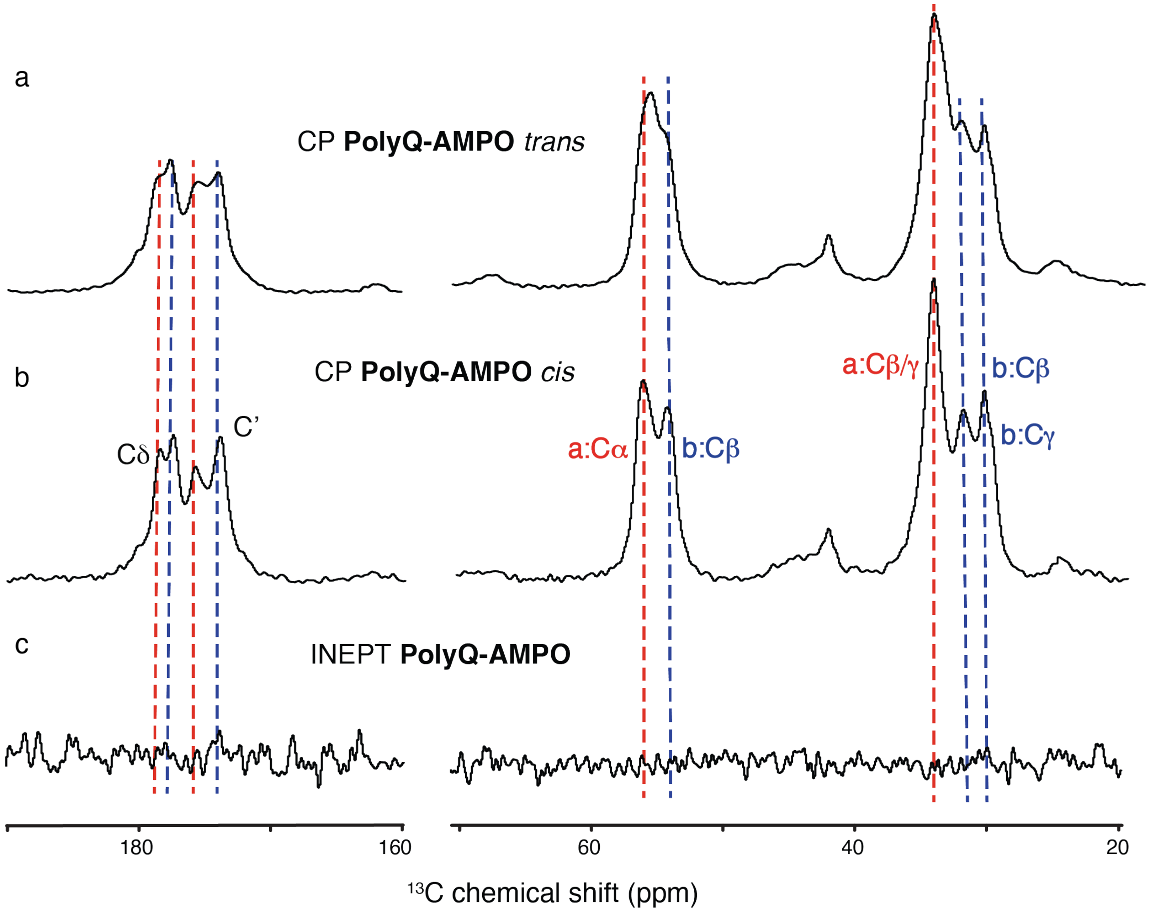

Figure S11. 1D ^{13}C CP and INEPT MAS ssNMR experiments on unlabeled **polyQ-AMPO** aggregates. a) 1D CP spectrum of aggregated *trans* isomer, (b) 1D CP spectrum of aggregated *cis* isomer and (c) 1D INEPT spectrum of the aggregated *trans* isomer. The lack of signal in panel c shows that no flexible residues can be detected, nor any significant portion of soluble monomers. The previously assigned ^{13}C peak positions for each of the two conformers of polyQ amyloid are marked with red (form a) and blue (form b) dashed lines.

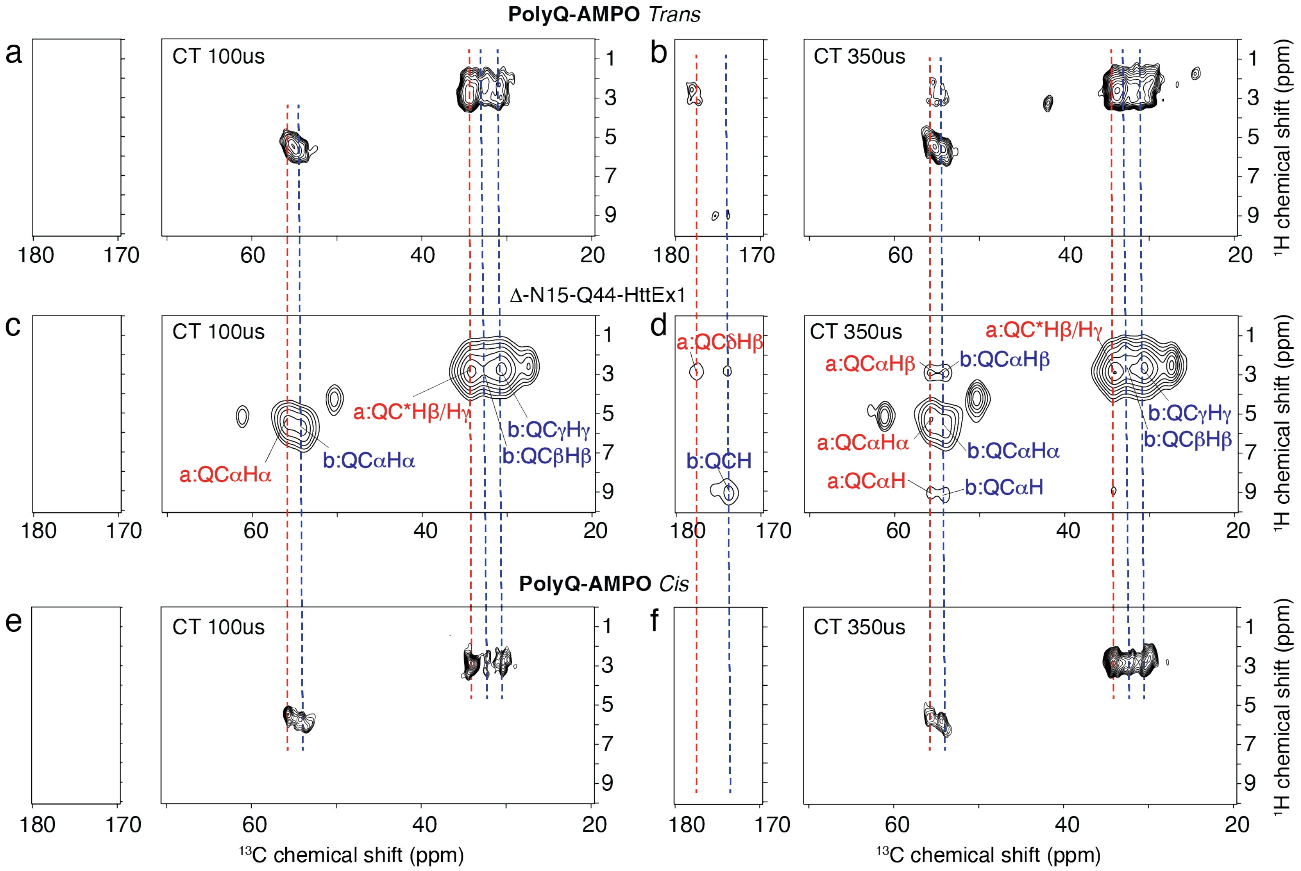

Figure S12. 2D ^{1}H-^{13}C HETCOR MAS ssNMR experiments. a) 2D HETCOR spectrum with CP contact time of 100 μs on aggregates formed by unlabeled **polyQ-AMPO** *trans*. b) 2D HETCOR spectrum with a CP contact time of 350 μs on aggregates formed by unlabeled **polyQ-AMPO** in *trans* configuration. c) 2D HETCOR spectrum with a CP contact time of 100 μs on aggregates formed by fully ^{13}C,^{15}N-labeled Δ-N15-Q44-HttEx1 fibrils.[10] d) 2D HETCOR spectrum with a CP contact time of 350 μs on the same Δ-N15-Q44-HttEx1 fibrils.[10] e) 2D HETCOR spectrum with CP contact time of 100 μs on aggregates formed by unlabeled **polyQ-AMPO** *cis*. f) Same as panel e, but with a contact time of 350 μs. The type "a" and "b" ssNMR signals characteristic of polyQ amyloid structures are indicated in panels c-d, and marked with red and blue lines respectively. The 100 μs spectra are expected to mostly show directly bonded H-C contacts, whereas the 350 μs spectra show also longer range transfers. Note that multiple narrower and distinct peaks are seen for the *cis* aggregates, compared to broader spectral features seen for the *trans* version. This is indicative of a greater degree of structural homogeneity in the former aggregates.

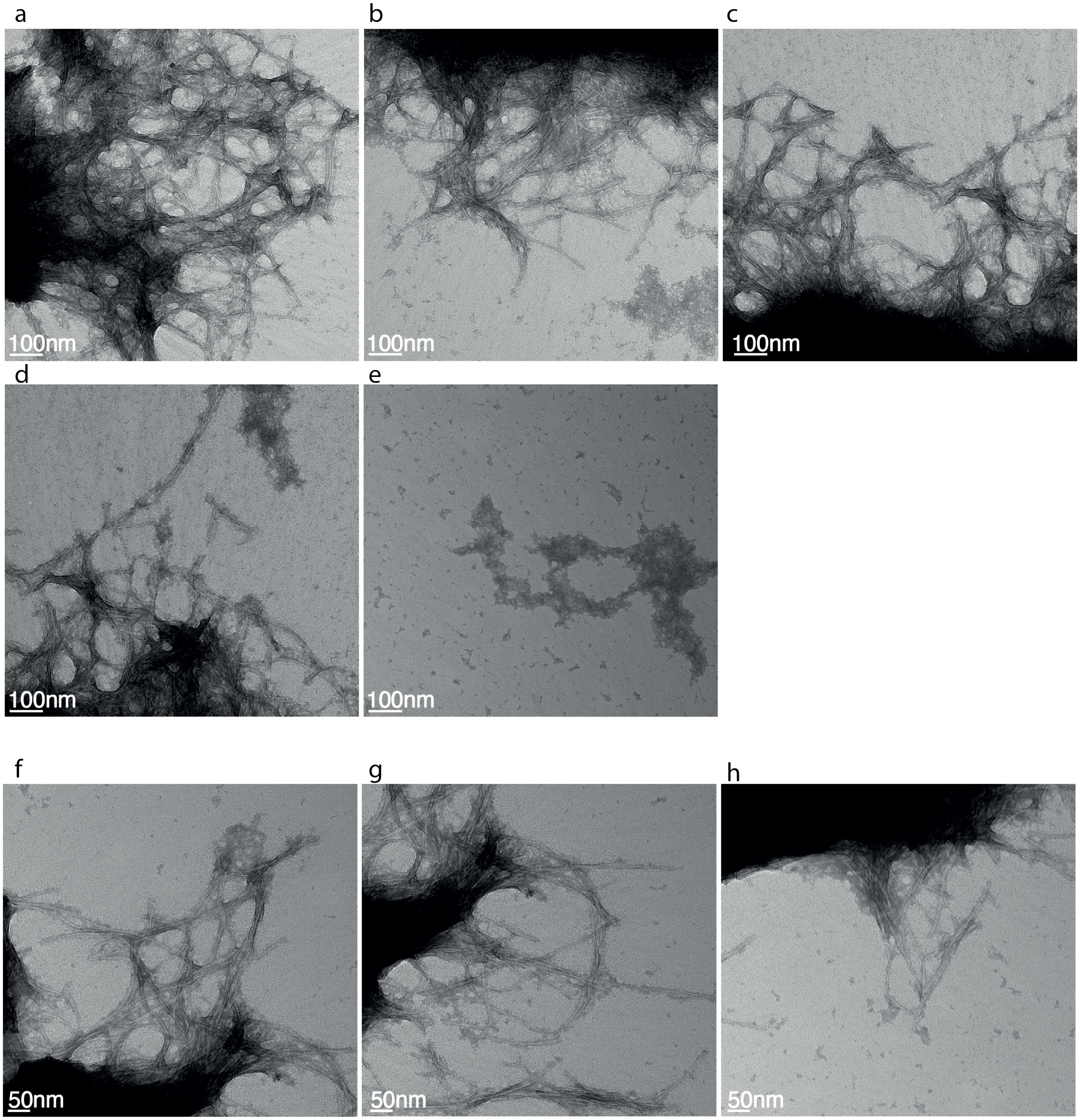

Figure S13. TEM data on *trans* **polyQ-AMPO** at two levels of magnification (45000x and 60000x). (a-e) Negative stain TEM of *trans* **polyQ-AMPO** fibrils formed at RT in PBS, at the magnification level of 45000x. (f-h) Negative stain TEM of *trans* **polyQ-AMPO** fibrils formed at RT in PBS, at the magnification level of 60000x. Note the extensive fibril bundling that is commonly seen for polyQ amyloid fibrils.

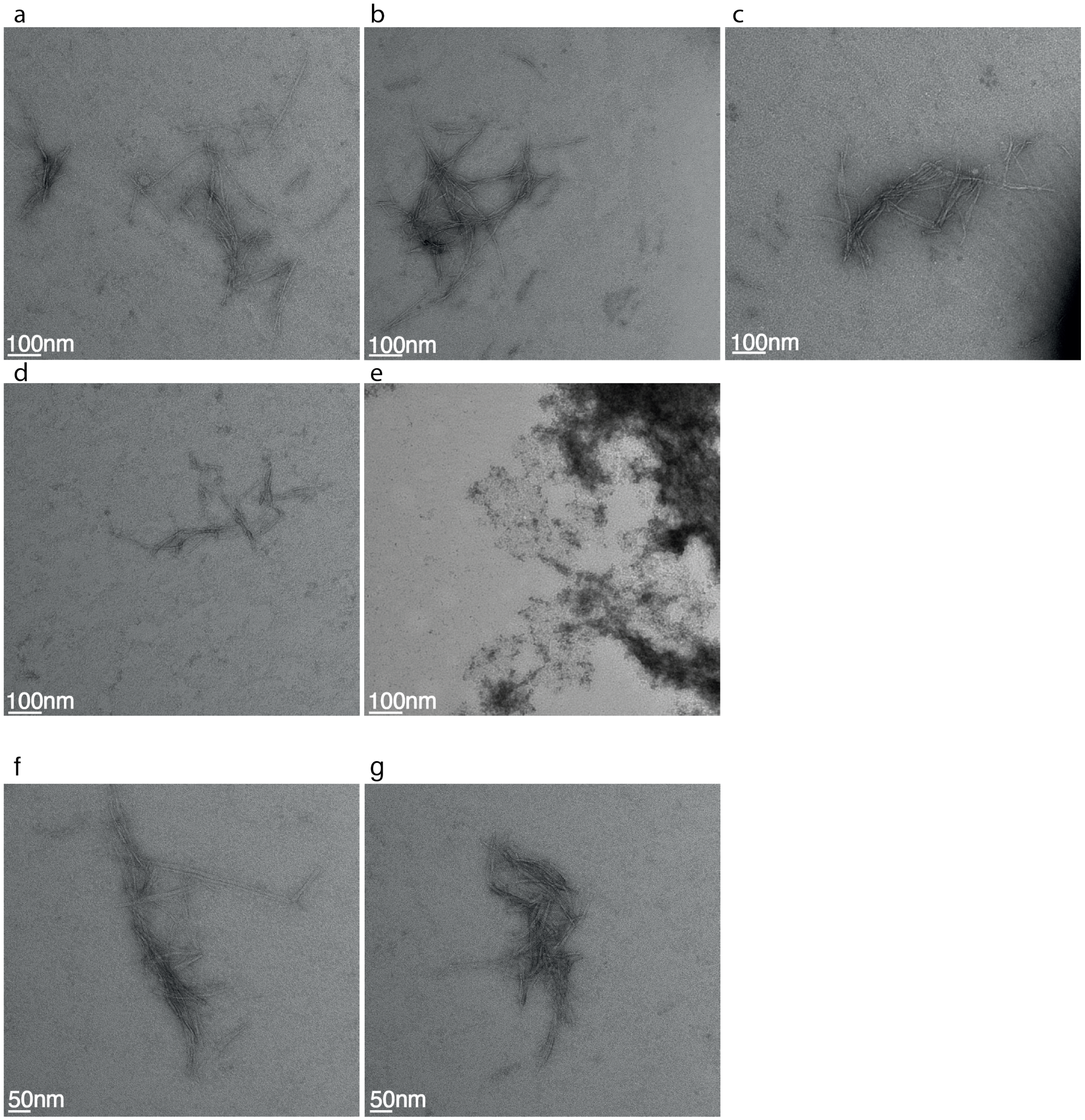

Figure S14. TEM data on *cis* **polyQ-AMPO** configuration two levels of magnification (45000x and 60000x). (a-e) Negative stain TEM of *cis* **polyQ-AMPO** fibrils formed at RT in PBS, at the magnification level of 45000x. (f-h) Negative stain TEM of *cis* **polyQ-AMPO** fibrils formed at RT in PBS, at the magnification level of 60000x.

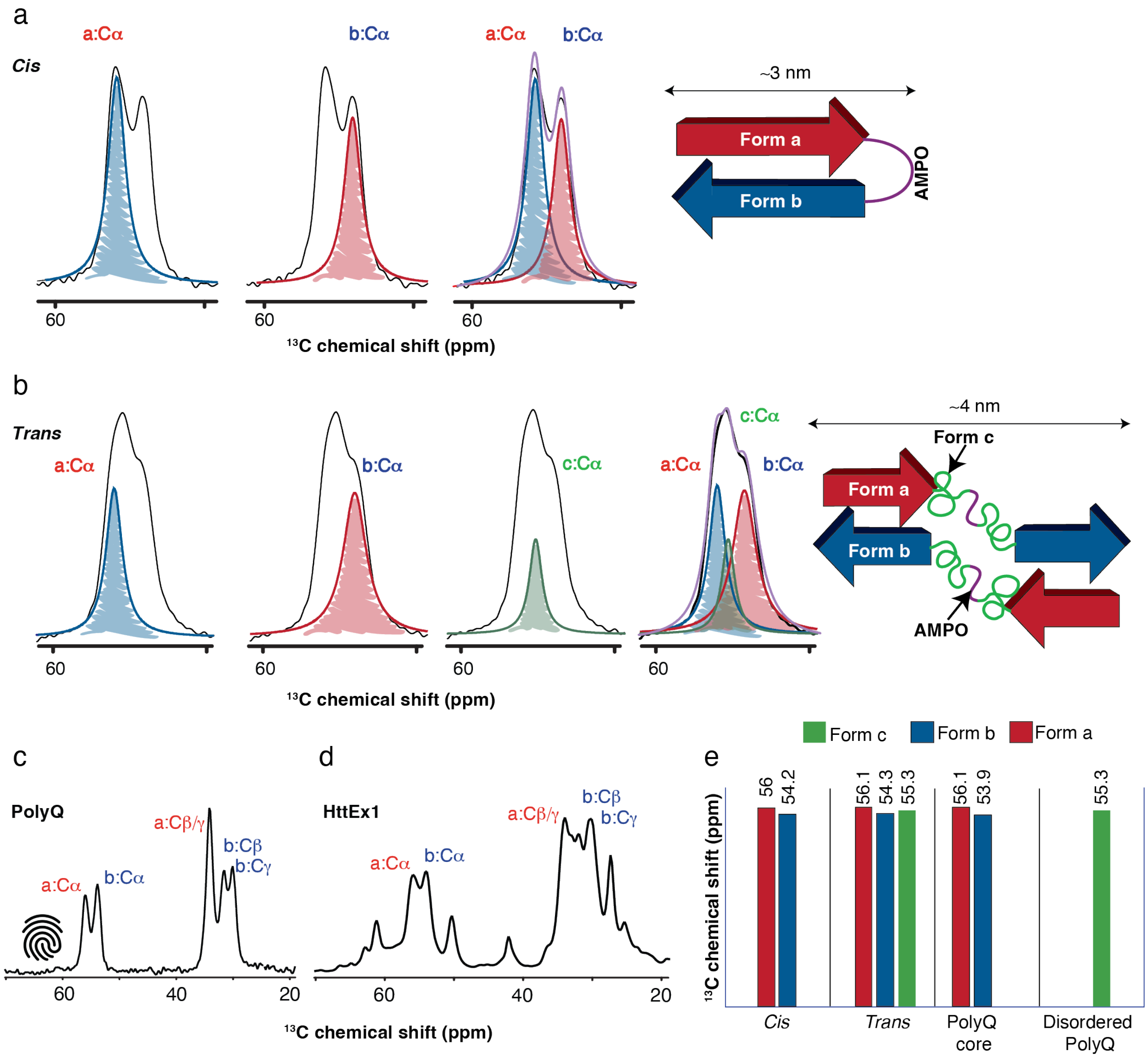

Figure S15. Spectral analysis showing how the different glutamine conformations modify the polyQ core ssNMR fingerprint in both *cis* and *trans* isomer aggregates. a) Spectral deconvolution of *cis* sample and how both "a" and "b" forms of the glutamine largely explain the two peaks in the carbon alpha region of the *cis* isomer. Form "a" and "b" are colored in red and blue respectively b) Spectral deconvolution of *trans* sample and how the three forms "a", "b" and "c" contribute to the carbon alpha peak in the *trans* isomer. Form "a", "b" and "c" are shown in red, blue and green color. c) PolyQ peptide spectrum of the Cα and Cβ region, showing the glutamine peaks in the two conformations: form "a" (red) and "b" (blue). The two peaks are highly reproducible and are considered the fingerprint of the polyglutamine core in huntingtin fibrils. d) Spectrum of Huntingtin Exon 1 (HttEx1) fibrils with labels for the two glutamine conformations. Unmarked peaks stem from other residue types in the protein. e) Bar graph comparing the chemical shift values used for fitting the **polyQ-AMPO** spectra to previously reported values for glutamine inside and outside the polyQ amyloid core. The chemical shift value of the disordered polyQ was found in reference [27]. The spectra deconvolution is performed with the program dmfit,[28] and the purple line (panel a and b) represents the fitting of the deconvolution.

3. Analytical data of all synthesized compounds

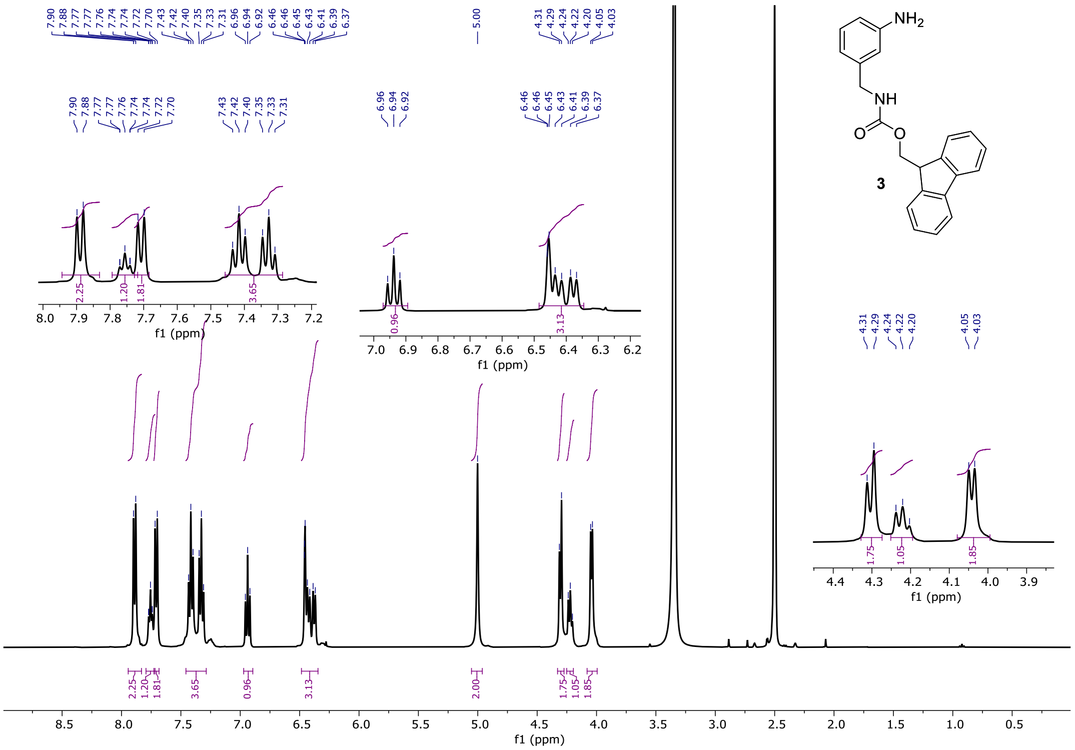

Figure S16. ^{1}H NMR (400 MHz) of compound **3** in DMSO-d_6.

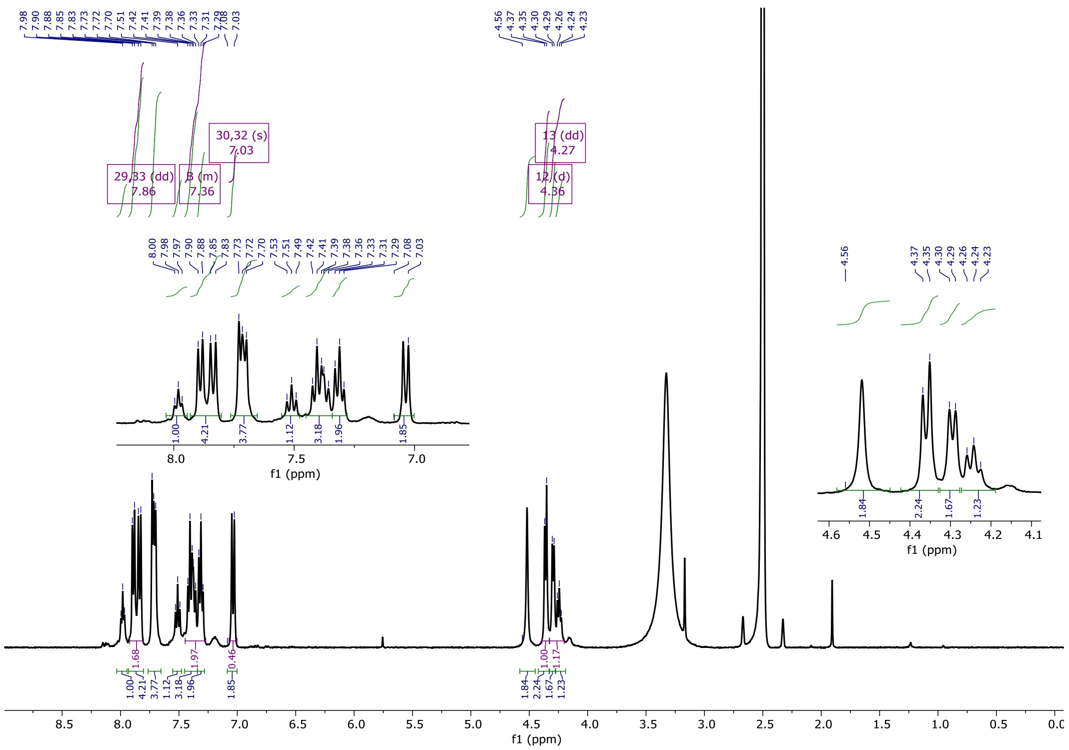

Figure S17. ^{1}H NMR (400 MHz) of compound **Fmoc-AMPO** in DMSO-d_6.

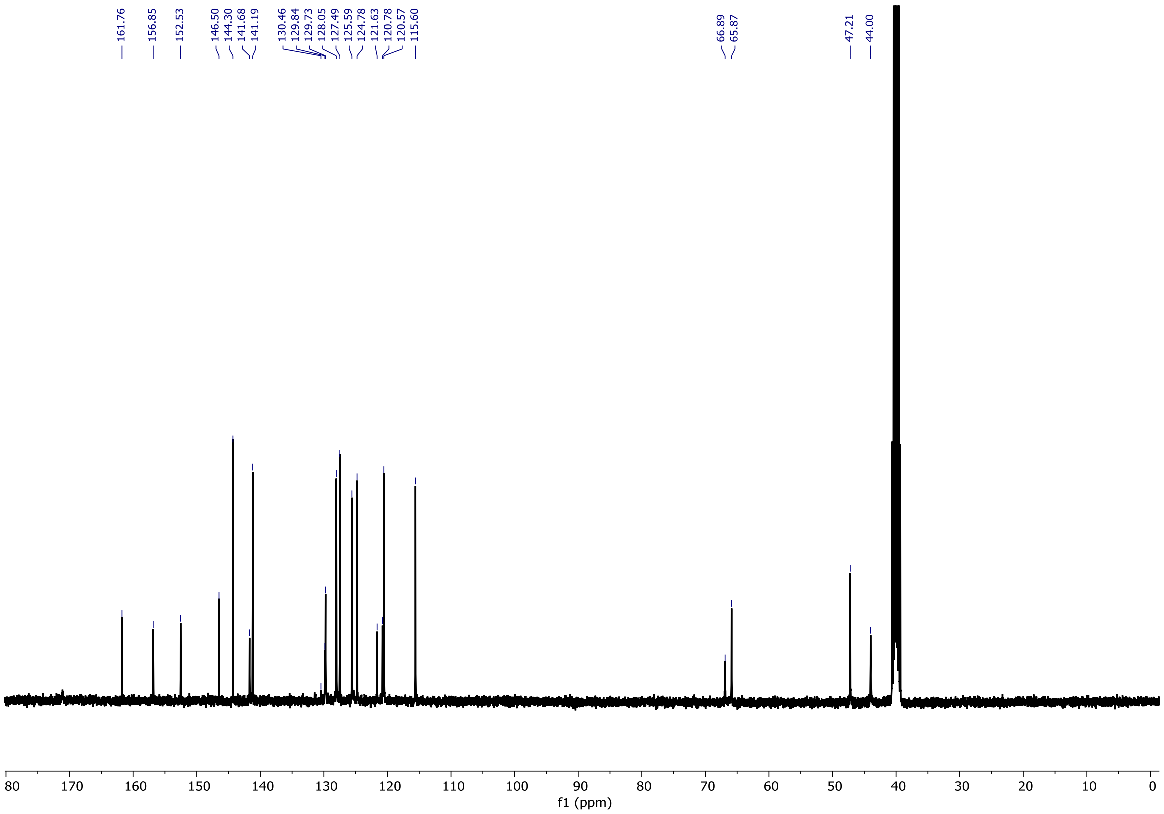

Figure S18. ^{13}C NMR (600 MHz) of compound **Fmoc-AMPO** in DMSO-d_6.

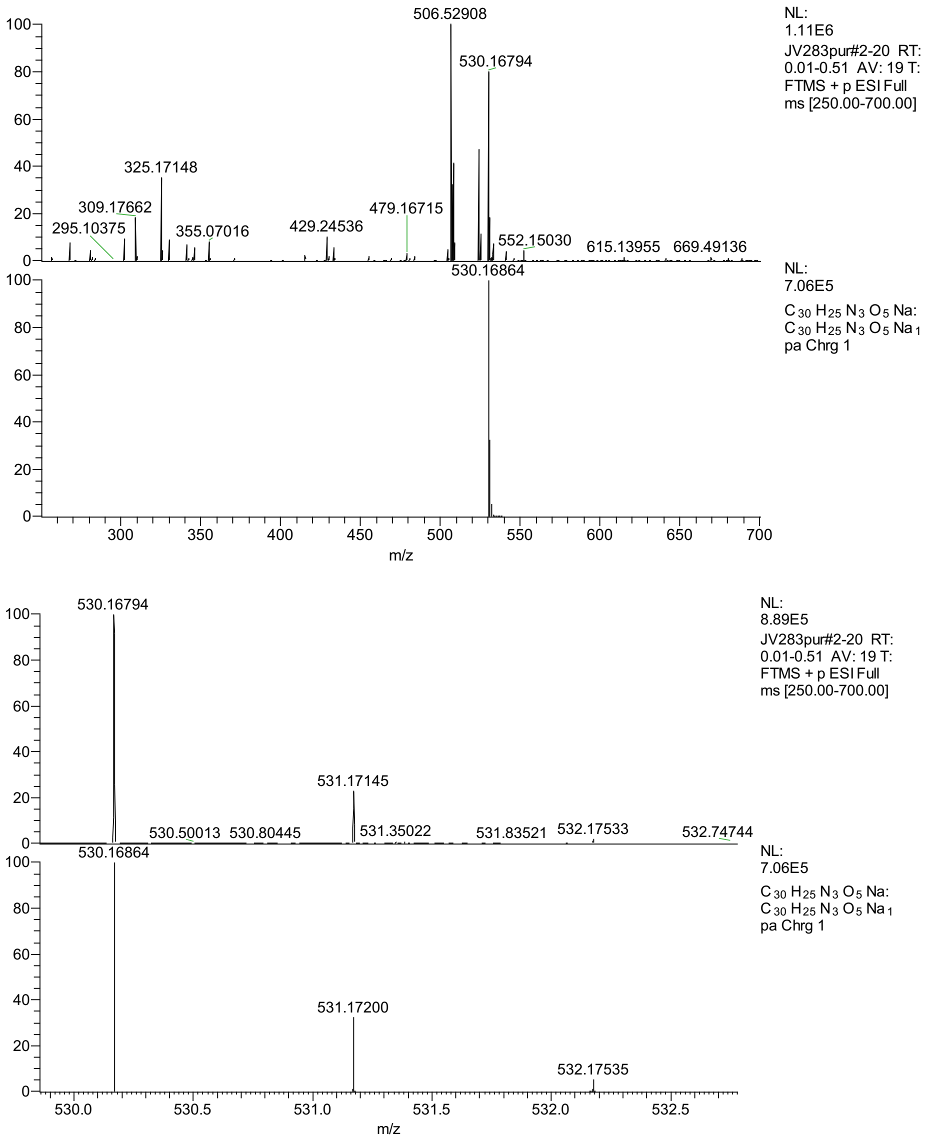

Figure S19. HRMS (ESI+) analysis for compound **Fmoc-AMPO**.

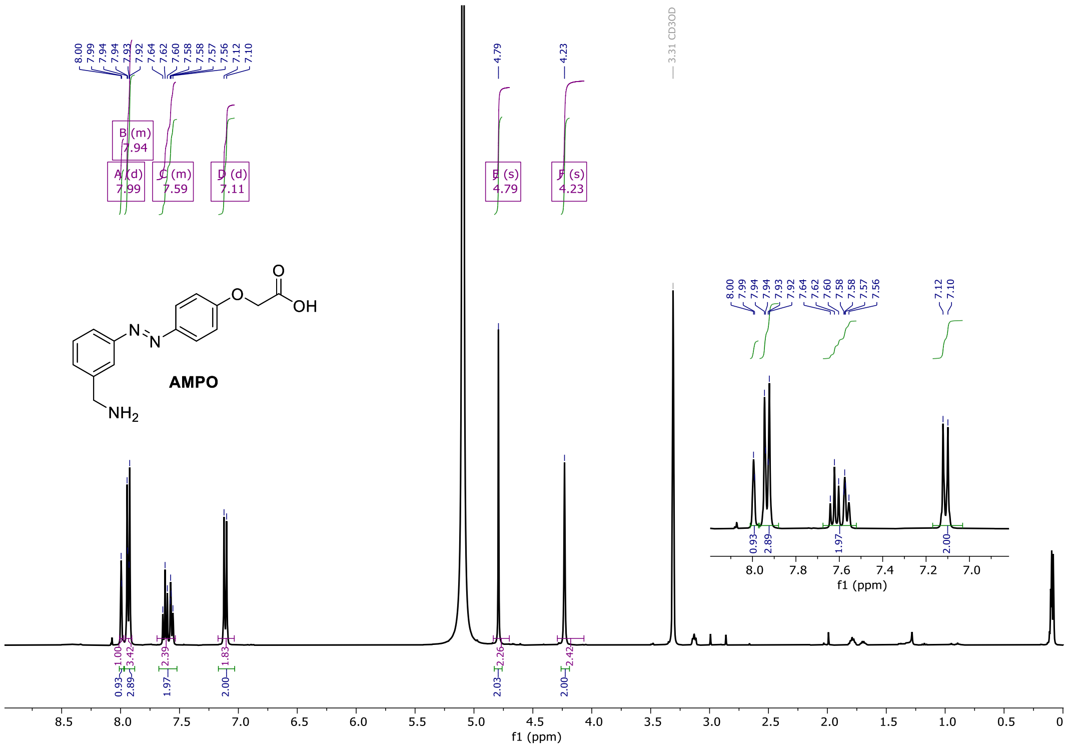

Figure S20. ^{1}H NMR (400 MHz) of compound **AMPO** in DMSO-*d*6.

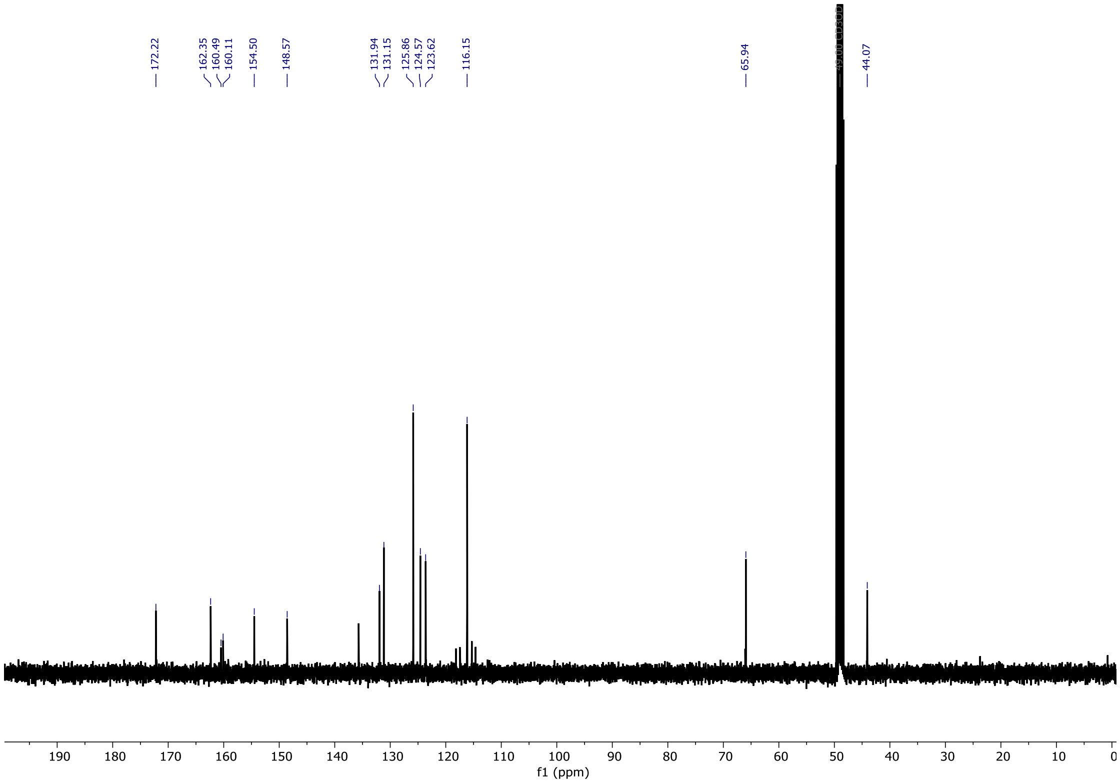

Figure S21. ^{13}C NMR (600 MHz) of compound **AMPO** in DMSO-*d*6.

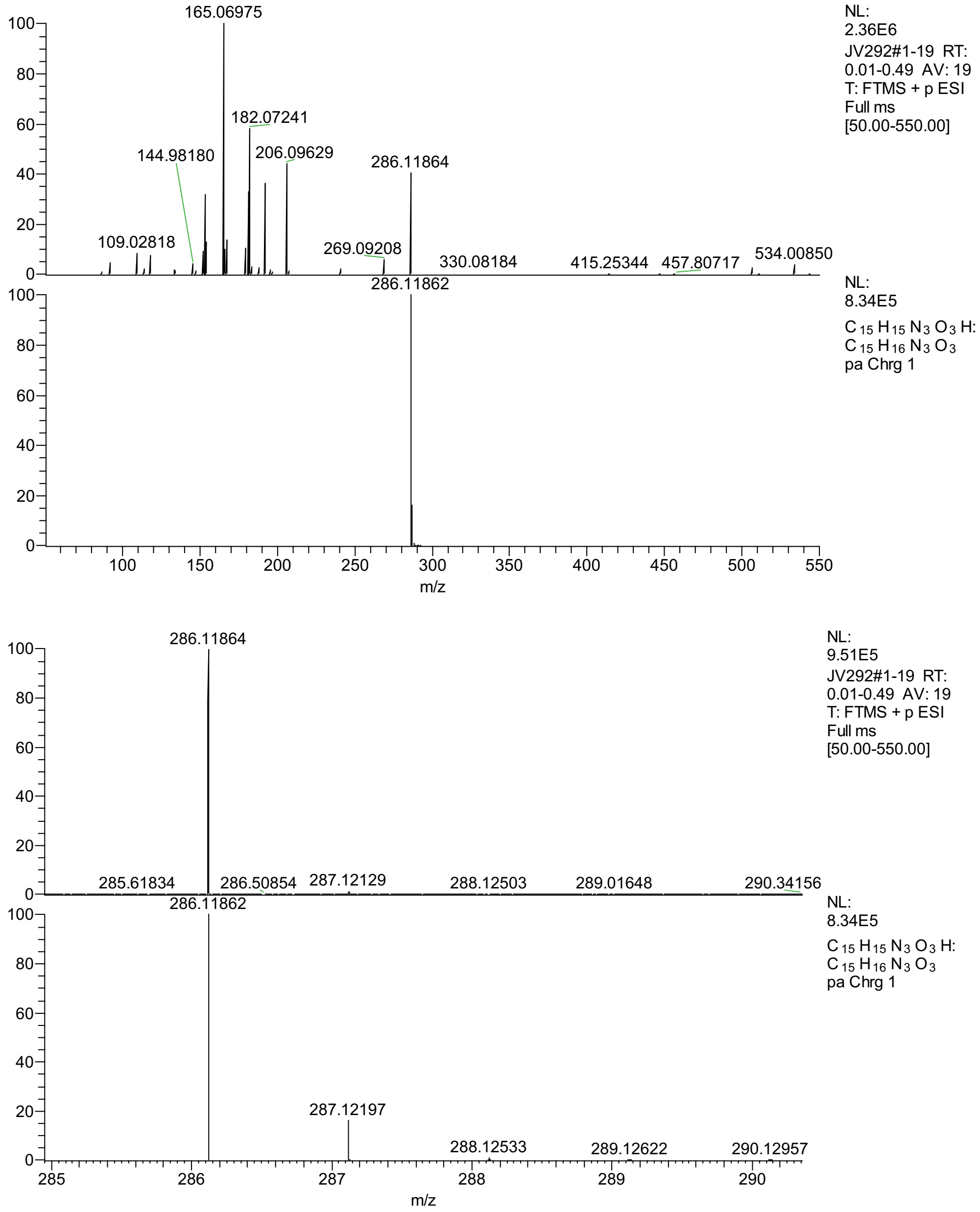

Figure S22. HRMS (ESI+) analysis for compound **AMPO**.

4. References cited in the Supporting Information

(1) Ivanova, T.; Hardes, K.; Kallis, S.; Dahms, S. O.; Than, M. E.; Künzel, S.; Böttcher-Friebertshäuser, E.; Lindberg, I.; Jiao, G.-S.; Bartenschlager, R.; Steinmetzer, T. Optimization of Substrate-Analogue Furin Inhibitors. *ChemMedChem* **2017**, *12* (23), 1953–1968. https://doi.org/10.1002/cmdc.201700596.

(2) Aemissegger, A.; Kräutler, V.; van Gunsteren, W. F.; Hilvert, D. A Photoinducible β-Hairpin. *J Am Chem Soc* **2005**, *127* (9), 2929–2936. https://doi.org/10.1021/ja0442567.

(3) Doran, T. M.; Nilsson, B. L. Incorporation of an Azobenzene β-Turn Peptidomimetic into Amyloid-β to Probe Potential Structural Motifs Leading to β-Sheet Self-Assembly. *Methods Mol Biol* **2018**, *1777*, 387–406. https://doi.org/10.1007/978-1-4939-7811-3_25.

(4) O'Nuallain, B.; Thakur, A. K.; Williams, A. D.; Bhattacharyya, A. M.; Chen, S.; Thiagarajan, G.; Wetzel, R. Kinetics and Thermodynamics of Amyloid Assembly Using a High-Performance Liquid Chromatography–Based Sedimentation Assay. In *Methods in Enzymology*; Academic Press, 2006; Vol. 413, pp 34–74. https://doi.org/10.1016/S0076-6879(06)13003-7.

(5) Kolarski, D.; Szymanski, W.; Feringa, B. L. Two-Step, One-Pot Synthesis of Visible-Light-Responsive 6-Azopurines. *Org Lett* **2017**, *19* (19), 5090–5093. https://doi.org/10.1021/acs.orglett.7b02361.

(6) Stranius, K.; Börjesson, K. Determining the Photoisomerization Quantum Yield of Photoswitchable Molecules in Solution and in the Solid State. *Sci Rep* **2017**, *7* (1), 41145. https://doi.org/10.1038/srep41145.

(7) Mandal, A.; Boatz, J. C.; Wheeler, T. B.; van der Wel, P. C. A. On the Use of Ultracentrifugal Devices for Routine Sample Preparation in Biomolecular Magic-Angle-Spinning NMR. *Journal of Biomolecular NMR 2017 67:3* **2017**, *67* (3), 165–178. https://doi.org/10.1007/S10858-017-0089-6.

(8) Morcombe, C. R.; Zilm, K. W. Chemical Shift Referencing in MAS Solid State NMR. *Journal of Magnetic Resonance* **2003**, *162* (2), 479–486. https://doi.org/10.1016/S1090-7807(03)00082-X.

(9) Bennett, A. E.; Rienstra, C. M.; Auger, M.; Lakshmi, K. V.; Griffin, R. G. Heteronuclear Decoupling in Rotating Solids. *J Chem Phys* **1995**, *103* (16), 6951–6958. https://doi.org/10.1063/1.470372.

(10) Boatz, J. C.; Piretra, T.; Lasorsa, A.; Matlahov, I.; Conway, J. F.; van der Wel, P. C. A. Protofilament Structure and Supramolecular Polymorphism of Aggregated Mutant Huntingtin Exon 1. *J Mol Biol* **2020**, *432* (16), 4722–4744. https://doi.org/10.1016/j.jmb.2020.06.021.

(11) van Rossum, B.-J.; Förster, H.; de Groot, H. J. M. High-Field and High-Speed CP-MAS13C NMR Heteronuclear Dipolar-Correlation Spectroscopy of Solids with Frequency-Switched Lee–Goldburg Homonuclear Decoupling. *Journal of Magnetic Resonance* **1997**, *124* (2), 516–519. https://doi.org/10.1006/jmre.1996.1089.

(12) Bielecki, A.; Kolbert, A. C.; De Groot, H. J. M.; Griffin, R. G.; Levitt, M. H. Frequency-Switched Lee—Goldburg Sequences in Solids. In *Advances in Magnetic and Optical Resonance*; Academic Press, 1990; Vol. 14, pp 111–124. https://doi.org/10.1016/B978-0-12-025514-6.50011-3.

(13) Delaglio, F.; Grzesiek, S.; Vuister, G. W.; Zhu, G.; Pfeifer, J.; Bax, A. NMRPipe: A Multidimensional Spectral Processing System Based on UNIX Pipes. *J Biomol NMR* **1995**, *6* (3), 277–293. https://doi.org/10.1007/BF00197809.

(14) Schneider, C. A.; Rasband, W. S.; Eliceiri, K. W. NIH Image to ImageJ: 25 Years of Image Analysis. *Nature Methods 2012 9:7* **2012**, *9* (7), 671–675. https://doi.org/10.1038/nmeth.2089.

(15) Miettinen, M. S.; Monticelli, L.; Nedumpully-Govindan, P.; Knecht, V.; Ignatova, Z. Stable Polyglutamine Dimers Can Contain β-Hairpins with Interdigitated Side Chains—But Not α-Helices, β-Nanotubes, β-Pseudohelices, or Steric Zippers. *Biophys J* **2014**, *106* (8), 1721–1728. https://doi.org/10.1016/j.bpj.2014.02.027.

(16) Helabad, M. B.; Matlahov, I.; Daldrop, J. O.; Jain, G.; Wel, P. C. A. van der; Miettinen, M. S. Integrative Determination of the Atomic Structure of Mutant Huntingtin Exon 1 Fibrils from Huntington's Disease. *bioRxiv* **2023**, 2023.07.21.549993. https://doi.org/10.1101/2023.07.21.549993.

(17) M.J. Frisch; G.W. Trucks; H.B. Schlegel; G.E. Scuseria; M.A. Robb; J.R. Cheeseman; J.A. Montgomery; Jr.T Vreven; K.N. Kudin; J.C. Burant; J.M. Millam; S.S. Iyengar; J. Tomasi; V.

Barone; B. Mennucci; M. Cossi; G. Scalmani; N. Rega; G.A. Petersson; H. Nakatsuji; M. Hada; M. Ehara; K. Toyota; R. Fukuda; J. Hasegawa; M. Ishida; T. Nakajima; Y. Honda; O. Kitao; H. Nakai; M. Klene; X. Li; J.E Knox; H.P. Hratchian; J.B. Cross; V. Bakken; C. Adamo; J. Jaramillo; R. Gomperts; R.E. Stratmann; O. Yazyev; A.J. Austin; R. Cammi; C. Pomelli; J.W. Ochterski; P.Y. Ayala; K. Morokuma; G.A. Voth; P. Salvador; J.J. Dannenberg; V.G. Zakrzewski; S. Dapprich; A.D. Daniels; M.C. Strain; O. Farkas; D.K. Malick; A.D. Rabuck; K. Raghavachari; J.B. Foresman; J.V. Ortiz; Q. Cui; A.G Baboul; S. Clifford; J. Cioslowski; B.B. Stefanov; G. Liu; A. Liashenko; P. Piskorz; I. Komaromi; R.L. Martin; D.J. Fox; T. Keith; M.A. AL-Laham; C.Y. Peng; A. Nanayakkara; M. Challacombe; P.M.W. Gill; B. Johnson; W. Chen; M.W. Wong; C. Gonzalez; J.A. Pople. GAUSSIAN 03. *Gaussian, Wallingford, CT.* 2004.

(18) Behrendt, R.; Renner, C.; Schenk, M.; Wang, F.; Wachtveitl, J.; Oesterhelt, D.; Moroder, L. Photomodulation of the Conformation of Cyclic Peptides with Azobenzene Moieties in the Peptide Backbone. *Angewandte Chemie International Edition* **1999**, *38* (18), 2771–2774. https://doi.org/10.1002/(SICI)1521-3773(19990917)38:18<2771::AID-ANIE2771>3.0.CO;2-W.

(19) Murawska, G. M.; Poloni, C.; Simeth, N. A.; Szymanski, W.; Feringa, B. L. Comparative Study of Photoswitchable Zinc-Finger Domain and AT-Hook Motif for Light-Controlled Peptide–DNA Binding. *Chemistry – A European Journal* **2019**, *25* (19), 4965–4973. https://doi.org/10.1002/chem.201900090.

(20) Doran, T. M.; Anderson, E. A.; Latchney, S. E.; Opanashuk, L. A.; Nilsson, B. L. An Azobenzene Photoswitch Sheds Light on Turn Nucleation in Amyloid-β Self-Assembly. *ACS Chem Neurosci* **2012**, *3* (3), 211–220. https://doi.org/10.1021/cn2001188.

(21) Podewin, T.; Rampp, M. S.; Turkanovic, I.; Karaghiosoff, K. L.; Zinth, W.; Hoffmann-Röder, A. Photocontrolled Chignolin-Derived β-Hairpin Peptidomimetics. *Chemical Communications* **2015**, *51* (19), 4001–4004. https://doi.org/10.1039/c4cc10304a.

(22) Dong, S.-L.; Löweneck, M.; Schrader, T. E.; Schreier, W. J.; Zinth, W.; Moroder, L.; Renner, C. A Photocontrolled β-Hairpin Peptide. *Chemistry - A European Journal* **2006**, *12* (4), 1114–1120. https://doi.org/10.1002/chem.200500986.

(23) Cattani-Scholz, A.; Renner, C.; Cabrele, C.; Behrendt, R.; Oesterhelt, D.; Moroder, L. Photoresponsive Cyclic Bis(Cysteinyl)Peptides as Catalysts of Oxidative Protein Folding

Angewandte Chemie International Edition **2002**, *41* (2), 289. https://doi.org/10.1002/1521-3773(20020118)41:2<289::AID-ANIE289>3.0.CO;2-2.

(24) Nuti, F.; Gellini, C.; Larregola, M.; Squillantini, L.; Chelli, R.; Salvi, P. R.; Lequin, O.; Pietraperzia, G.; Papini, A. M. A Photochromic Azobenzene Peptidomimetic of a β-Turn Model Peptide Structure as a Conformational Switch. *Front Chem* **2019**, *7* (MAR), 1–9. https://doi.org/10.3389/fchem.2019.00180.

(25) Albert, L.; Xu, J.; Wan, R.; Srinivasan, V.; Dou, Y.; Vázquez, O. Controlled Inhibition of Methyltransferases Using Photoswitchable Peptidomimetics: Towards an Epigenetic Regulation of Leukemia. *Chem Sci* **2017**, *8* (6), 4612–4618. https://doi.org/10.1039/C7SC00137A.

(26) Deeg, A. A.; Schrader, T. E.; Strzalka, H.; Pfizer, J.; Moroder, L.; Zinth, W. Amyloid-Like Structures Formed by Azobenzene Peptides: Light-Triggered Disassembly. *Spectroscopy: An International Journal* **2012**, *27*, 387–391. https://doi.org/10.1155/2012/108959.

(27) Matlahov, I.; Boatz, J. C.; Van Der Wel, P. C. A. Selective Observation of Semi-Rigid Non-Core Residues in Dynamically Complex Mutant Huntingtin Protein Fibrils. *J Struct Biol X* **2022**, *6*, 100077. https://doi.org/10.1016/j.yjsbx.2022.100077.

(28) Massiot, D.; Fayon, F.; Capron, M.; King, I.; Le Calvé, S.; Alonso, B.; Durand, J. O.; Bujoli, B.; Gan, Z.; Hoatson, G. Modelling One- and Two-Dimensional Solid-State NMR Spectra. *Magnetic Resonance in Chemistry* **2002**, *40* (1), 70–76. https://doi.org/10.1002/MRC.984.
